# GI-SleepNet: A Highly Versatile Image-Based Sleep Classification Using a Deep Learning Algorithm

**DOI:** 10.3390/clockssleep3040041

**Published:** 2021-11-01

**Authors:** Tianxiang Gao, Jiayi Li, Yuji Watanabe, Chijung Hung, Akihiro Yamanaka, Kazumasa Horie, Masashi Yanagisawa, Masahiro Ohsawa, Kazuhiko Kume

**Affiliations:** 1 Department of Neuropharmacology, Graduate School of Pharmaceutical Sciences, Nagoya City University, Nagoya 467-8603, Japan; amakate-yaku@hotmail.com (T.G.); li_jiayi1890@126.com (J.L.); ohsawa@phar.nagoya-cu.ac.jp (M.O.); 2 Graduate School of Science, Nagoya City University, Nagoya 467-8501, Japan; yuji@nsc.nagoya-cu.ac.jp; 3 Department of Neuroscience II, Research Institute of Environmental Medicine, Nagoya University, Nagoya, 467-8603, Japan; sethhtes33@gmail.com (C.H.); yamank@riem.nagoya-u.ac.jp (A.Y.); 4 Graduate School of Systems and Information Engineering, University of Tsukuba, Tsukuba 305-8577, Japan; horie@bipl-sdnn.org; 5 Center for Computational Sciences, University of Tsukuba, Tsukuba 305-8577, Japan; 6 International Institute for Integrative Sleep Medicine, University of Tsukuba, Tsukuba 305-8577, Japan; yanagisawa.masa.fu@u.tsukuba.ac.jp

**Keywords:** EEG, sleep scoring, 2D-CNN, GANs, tiny dataset

## Abstract

Sleep-stage classification is essential for sleep research. Various automatic judgment programs, including deep learning algorithms using artificial intelligence (AI), have been developed, but have limitations with regard to data format compatibility, human interpretability, cost, and technical requirements. We developed a novel program called GI-SleepNet, generative adversarial network (GAN)-assisted image-based sleep staging for mice that is accurate, versatile, compact, and easy to use. In this program, electroencephalogram and electromyography data are first visualized as images, and then classified into three stages (wake, NREM, and REM) by a supervised image learning algorithm. To increase its accuracy, we adopted GAN and artificially generated fake REM sleep data to equalize the number of stages. This resulted in improved accuracy, and as little as one mouse’s data yielded significant accuracy. Due to its image-based nature, the program is easy to apply to data of different formats, different species of animals, and even outside sleep research. Image data can be easily understood; thus, confirmation by experts is easily obtained, even when there are prediction anomalies. As deep learning in image processing is one of the leading fields in AI, numerous algorithms are also available.

## 1. Introduction

Sleep is a stable systemic state that, in mammals, is controlled by homeostasis and endogenous circadian rhythms. Ascertaining how sleep is composed makes it easier and faster to evaluate and quantify sleep in research and clinical settings. Nowadays, it is widely known that sleep is composed of two parts, non-rapid eye movement (NREM) sleep and REM sleep. However, it took researchers nearly three decades, from 1924 to 1953, to separate these two types of sleep. In this process, long-term visual judgment by researchers played a key role. Sleep is characterized using an electroencephalogram (EEG), which was first developed by Hans Berger in 1924 [1]. He called the EEG a “brain mirror,” reflecting the “electrical psychic energy” within cortical tissue. He analyzed the wave phase patterns and described the α and β waves. Over a decade later, Alfred Loomis showed that human EEG patterns dramatically change from the wake to sleep stages [2]. Loomis initially classified sleep into five stages (A, B, C, D, and E), which are primarily manifested by the characteristic patterns of the α and spindle waves. Initially, the characterization and transition of brainwave frequencies were considered essential features. In his study, Loomis used the six-channel EEG of 30 s because the record sheets were automatically cut by a scissor every 30 s, and this marked the earliest conceptual origin of the classification epoch. These separate epochs were visually judged by researchers in a manner similar to the workflows conducted by modern polysomnography (PSG) technicians. For this reason, even now, most sleep classification algorithms use 30 s as one epoch length to determine the sleep stage.

After the rapid eye movement (REM) sleep stage was discovered by Aserinsky and Kleitman in 1953, electrooculography (EOG) and mentalis muscle electromyography (EMG) were used for sleep classification. Rechtschaffen and Kales then set up the PSG criteria in 1968 [3], which have been widely used to the present day with only minor modifications. However, without EOG, EMG, or the automatic integral calculation method being used for relative band powers, Loomis’s sleep classification criteria from the 1930s closely resembles the modern one, suggesting that EEG patterns play a more critical role than any other channel. In addition, visual judgments by technicians remain important for classifying sleep stages.

In addition to the human PSG, considerable demand exists for research on rodent sleep data. Rodents also have a sleep–wake cycle consisting of different sleep stages. However, there are some significant differences compared with humans. First, nocturnal rodents tend to get more sleep in the light period compared with humans, who sleep in the dark period. Second, unlike human sleep which is monophasic and repeats the NREM–REM sleep cycle (lasting about 90 min) three to six times successively only during night, the sleep of rodents is polyphasic and occurs both during the day and night time, and does not usually repeat the NREM–REM sleep cycle (lasting for several minutes to longer, irregular duration) successively. Third, the NREM sleep of rodents is not subdivided, unlike humans. Normally, all sleep states, excluding REM, are regarded as NREM. As a result, the rodent sleep cycle is relatively shorter, not continuous, fragmented, and unstable to external environmental changes. Nonetheless, rodents are a great model to understand human sleep, and a simple and reliable method to classify their sleep stage is required. In the specific representation of electrophysiology, the classification criteria of the sleep stages of mice are different from human PSG classification. The murine non-rapid eye movement (NREM) stage shows low EMG amplitude and high EEG δ-wave power, and NREM is classified as one stage without any further subdivision. The REM stage shows a higher θ-wave power than any other frequency band. Thus, three sleep stages, namely wake, NREM, and REM, have typical individual features on the EEG power spectrum. Researchers of murine sleep usually use an automatic scoring commercial software, such as SleepSign (Kissei Comtec Co. Ltd., Matsumoto, Japan) [4] or a MATLAB advanced toolbox such as EEGLAB [5]. However, these processing tools may present some obstacles for new researchers due to the cost or the requirement for high-level programming skills. Furthermore, due to the shorter sleep cycle and the relative unstableness of the sleep stage in mice, the one-epoch length is usually set shorter compared with humans, usually being shorter than 30 s.

Thanks to technical advances in machine learning, for the past 10 years we have had the opportunity to utilize artificial neural networks to study the sleep–wake cycle activities generated by natural neural networks. An unsupervised algorithm known as FASTER [6] (fully automated sleep staging method via EEG/EMG recordings) attained prominence even before the first TensorFlow (Mountain View, CA, USA) beta version was released in 2015. FASTER calculates the power spectrum of both EEG and EMG and performs a clustering of the power spectrum values using principal component analysis. The sensitivity performances of the NREM and wake states are comparatively fine. However, because the clustering of rare events (REM) for “hard” rule classical clustering analysis is complex, the sensitivity of REM is low and unstable in different experimental environments.

After TensorFlow was released, most of the algorithms were aimed at human PSG; however, later, these human-based approaches were found to be instructive for other mammalian sleep studies. In 2017, Guo et al. open-sourced the DeepSleepNet model for EEG single channel-based sleep-stage scoring [7], which was trained by the Sleep-EDF dataset for humans. Before DeepSleepNet, most classification methods were dependent on complex calculations for extracting band power features. However, the DeepSleepNet model works without utilizing any hand-engineered features by merging the two branches (EEG and EMG) of a convolutional neural network (CNN) and bidirectional long short-term memory (Bi-LSTM) cells.

Recently, MC-SleepNet was created for sleep-stage scoring in mice [8], inspired by DeepSleepNet with the addition of EMG. The performance analysis results, excluding the low precision of REM on small-scale datasets, revealed that MC-SleepNet was superior. However, for laboratory-level studies, particularly for some rare transgenic strains that are not easily propagated, performance on small-scale datasets is also important. This is also true of large-scale datasets, particularly for research related to REM sleep anomalies in mice [9]. The problem with the one-dimensional CNN is its weakness in outlier detection, especially when applied to sleep studies. This is considered to be a cause of DeepSleepNet’s low sensitivity for N1, as the N1 stage is short and contains various noises. The problem with MC-SleepNet is that it is very difficult for researchers to re-examine the data. This is because one-dimensional data needs to be visualized before examination. This inspired us to potentially visualize the data directly as two-dimensional (2D) to cut out the middle processes. Visualized data can certainly also be analyzed with CNNs.

In fact, the CNN was originally developed to analyze 2D image data. Thus far, 2D image-based CNN-LSTM networks have been applied very successfully in the field of medical diagnostic imaging [10,11]. This strategy has also been shown to be successful in the analysis of phenotypic convergence for the taxonomy of species such as butterflies [12]. An analysis of the features of the images extracted by a 2D CNN even showed that identifying the migration patterns between phases was possible. This type of parsing method can not only classify all discrete data, but it can also provide a visual interpretation of the transformations between various stages and the data relationships within each stage group.

For these reasons, we commenced this project. We hoped that we could develop an algorithm to achieve high accuracy and ease of use for automatic sleep classification in a cost-effective way, allowing researchers to enhance the analysis of sleep as a whole, rather than overly focusing on fragments within a large time scale. In particular, in order to overcome the problems of previous methods that were not easy-to-use, we developed a novel method called GI-SleepNet, a GAN-assisted image-based program, to process EEG and EMG data. As the first step, we produced an image file corresponding to each classification epoch, composed of an EEG power spectrum plot and an EMG raw wave data graph of that epoch. We then manually classified these epoch images into three stages—wake, NREM, and REM. Next, we used a 2D CNN for supervised learning of the designated images. GI-SleepNet precisely follows the logic and workflow of expert technicians, and allows for the easy identification of what the machine is learning. In addition, the accumulated knowledge and numerous methods on the deep learning of images are readily applicable.

## 2. Results

### 2.1. Data Image Production

**Figure 1:**
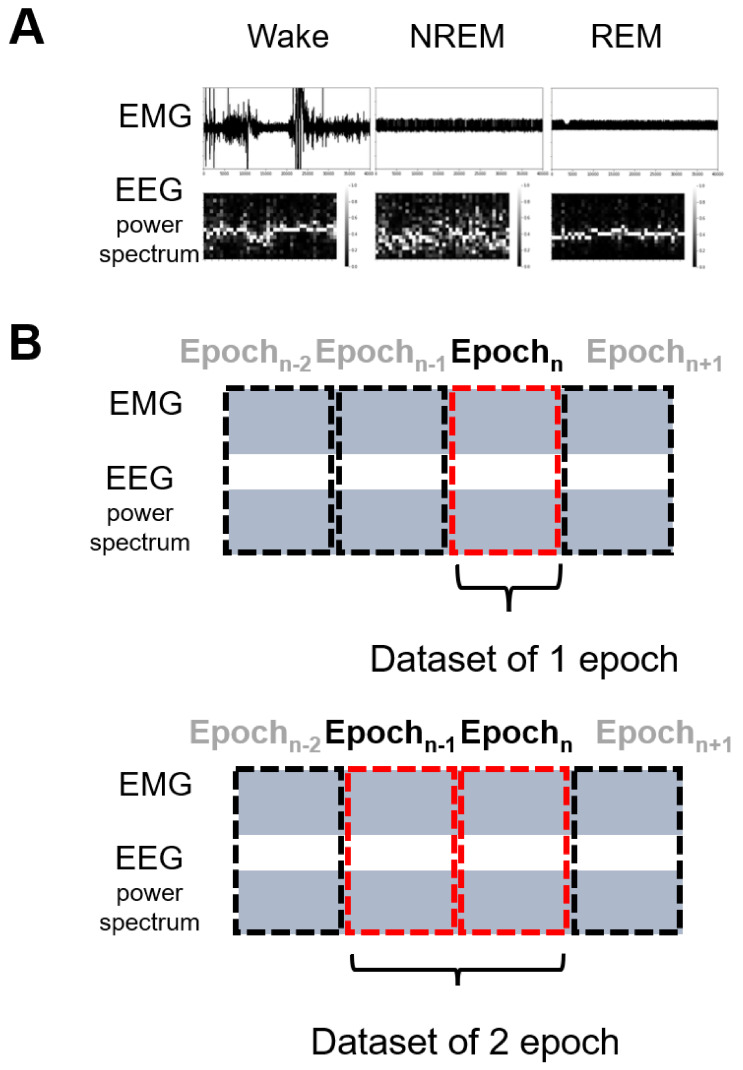
Image production for image-based machine learning. (**A**) Sample images of three sleep stages—wake, NREM, and REM. The upper part of the data image is the EMG. The vertical coordinate is fixed between all the images. The lower part is the heatmap of the EEG power spectrum (1–20 Hz) of 1 s bins. The brightness of the heatmap is normalized by Python’s scikit-learn library. (**B**) Schematic representation of 1- and 2-epoch data image generation. Images are labeled by the sleep stage and the 2-epoch image is classified according to the designation of the latter half of the 20-s epoch.

To develop an image-based process, we first determined the format of a single image. All images were generated using Python’s Matplotlib and Seaborn libraries. The upper and lower parts of a single data image were the EMG raw data graph and heatmap of the EEG power spectrum, respectively (Figure 1A). The latter was calculated by fast Fourier transform (FFT) and normalized by Python’s Scikit-learn library. The size of the original image was 800 × 800 pixels. The image had 32-bit color depth, although all the images were produced at gray scale. All of the marks on the horizontal and vertical coordinates, as well as the color bar of the heatmap, remained on the images, which helped with human visual perception and did not interfere with machine learning, as they were identical in all images. The values of both the horizontal and vertical coordinates were set to a constant between images in advance.

We created two image datasets with different data period lengths (Figure 1B). One contained one epoch (20 s) of EEG/EMG information, whereas the other contained two epochs (40 s) consisting of the epoch of interest and the preceding epoch. For machine learning, we scaled down the image size.

### 2.2. Selection of the Appropriate Network Structure from Pretrained Models

**Figure 2:**
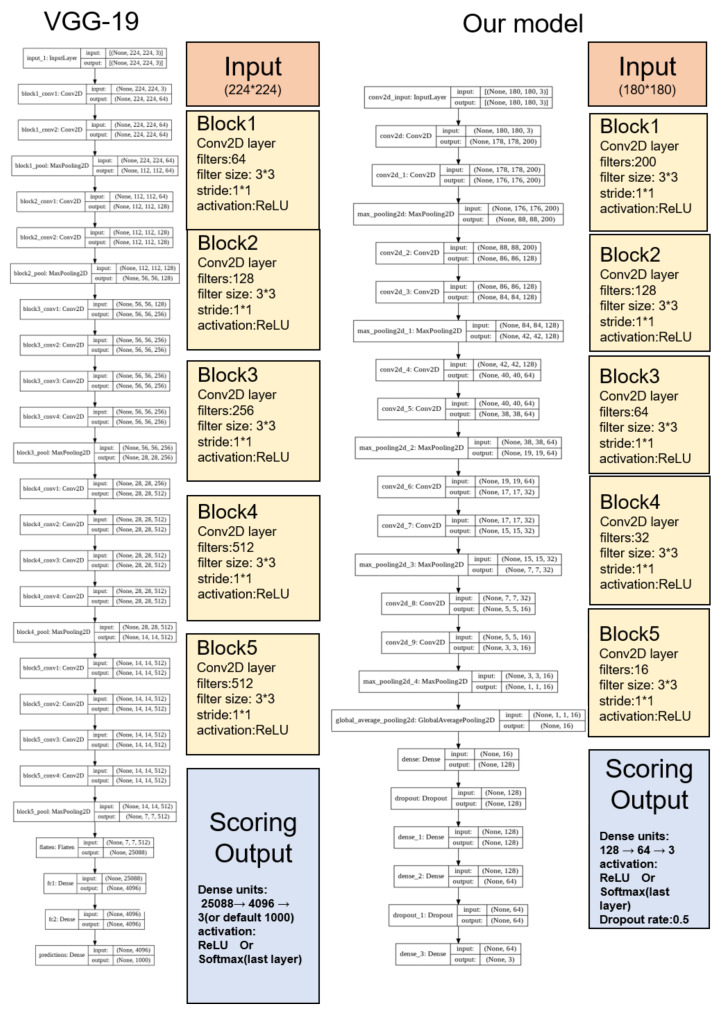
A modified network structure based on VGG-19. The low precision of REM using the existing algorithm is due to imbalanced multiclass classification sleep datasets. The ratio of the three stages of the ordinary mouse is approximately 10 : 10 : 1 (wake:NREM:REM) under the conventional experimental conditions. The too small sample size of the REM severely reduces the precision of REM, especially on a small-scale dataset [8], which needed to be resolved. Thus, we decided to increase the number of REM epochs.

For preliminary work, to confirm whether the sleep scoring using the created images worked effectively, we constructed our own small image dataset using EEG and EMG data from C57BL/6J mice. In this trial, the input size of the images was set to 800 × 800 pixels. After trying some transfer learning models such as DenseNet (accuracy = 53%), MobileNet (accuracy = 67%), and ResNet (accuracy = 78%) on our dataset, we found that VGG-19 (accuracy = 94%) had good potential. In order to reduce the amount of data to be calculated, we tried to reduce the input size and found that the performance could still be maintained at 180 × 180. The structure was quite similar to VGG-19 in that both have five blocks of 2D-CNN to extract the image information. We then added four dense layers and two dropout layers at the ends of the networks to prevent overfitting (Figure 2).

### 2.3. Expansion of the Dataset by GAN

The ratio of the three sleep stages of an ordinary mouse is approximately 10 : 10 : 1 (wake:NREM:REM) under conventional experimental conditions. Thus, we suspected that the low precision of REM using the existing algorithm was due to an imbalance in the number of stages in the sleep datasets. The small sample size of the REM may have reduced the precision, particularly on the small-scale dataset [8], which was a problem that needed to be solved. Thus, we decided to increase the number of REM epochs.

**Figure 3:**
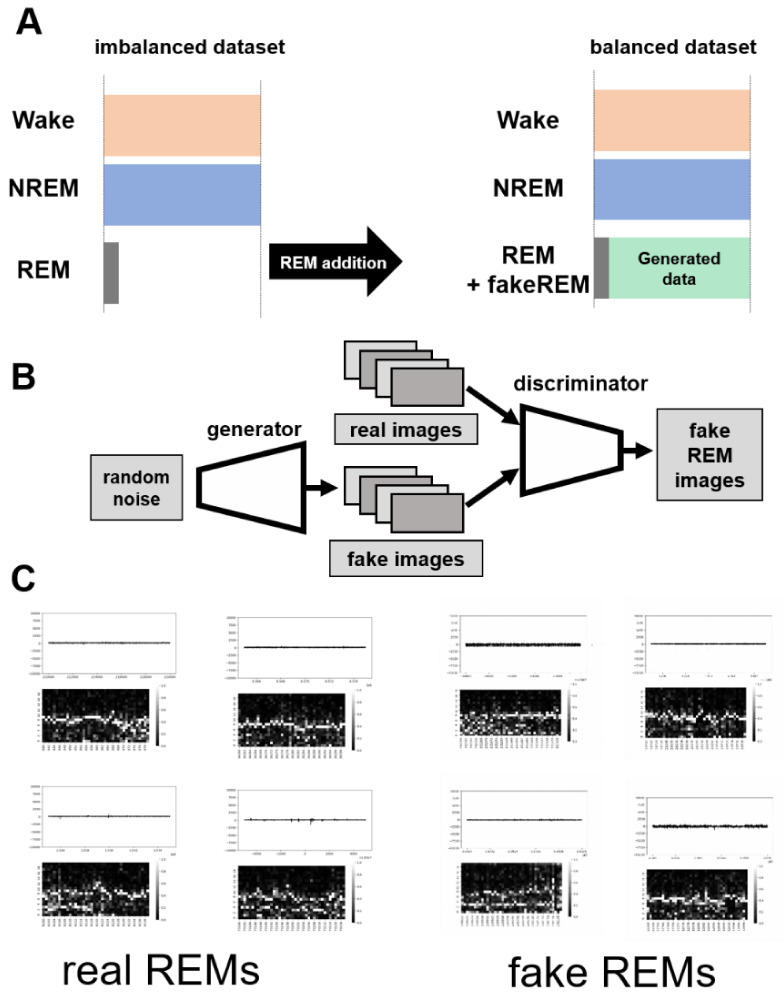
Expansion of the dataset using fake images. (**A**) Schematic representation of WGAN-GP-based image expansion. Bottom left shows the true image and the bottom right is the fake image generated based on the dataset. (**B**) Modified DCGAN (deep convolutional generative adversarial network) structure. High-resolution images (512 × 512) will be generated in our model. (**C**) Real REM sleep and fake REM images.

**Figure 4:**
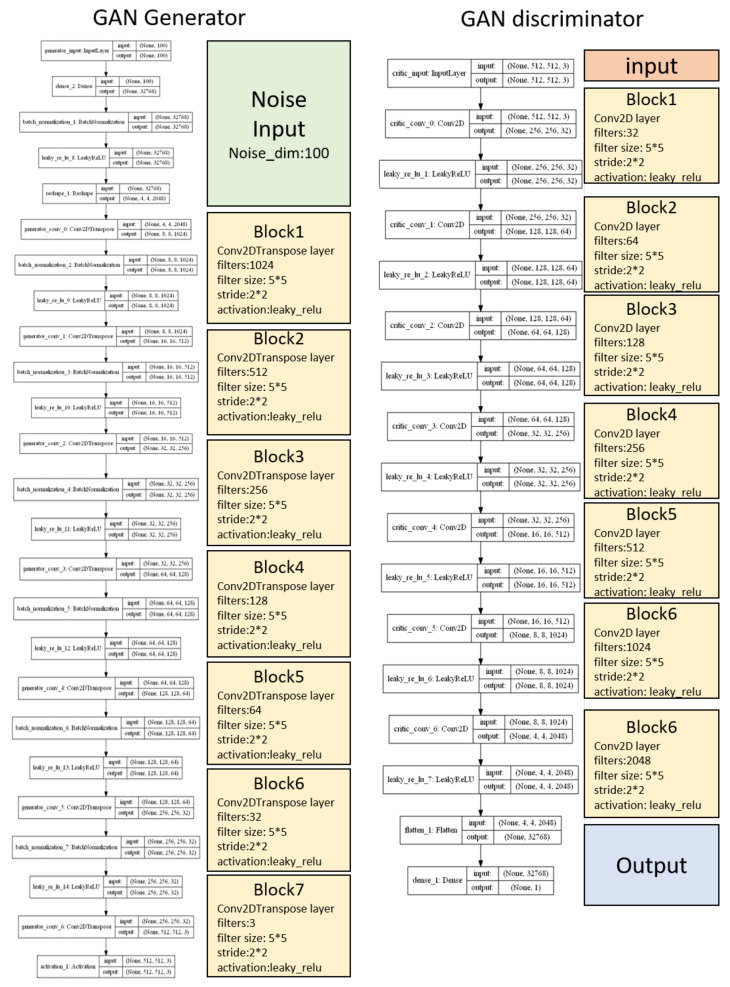
Generator and discriminator structure of our modified WGAN-GP.

Instead of increasing the size of the actual dataset, which is time-consuming and laborious, we increased the size of the REM epoch with artificially produced fake REM data by designing a REM data generator using a deep convolutional generative adversarial network (DCGAN) (Figure 3A,B). GANs for data augmentation with medical image data have been widely used [13]. As low-resolution images are difficult to check, we tried to improve the resolution of the generated image to 512 × 512 (Figure 3C). Since it is difficult for a standard DCGAN model to generate high-resolution images, we chose an advanced Wasserstein GAN with gradient penalty (WGAN-GP) model, which was originally described for the well-known CelebA face-dataset training in the O‘Reilly series book Generative Deep Learning (Chapter 4.6) [14]. The generator of WGAN-GP could be considered as a reverse version of our classifier. In this book, the original version had only five blocks with a 128 × 128 output size. We modified this structure and added another two blocks to enable it to accommodate our high-resolution output demand (Figure 4). Accordingly, we also improved the discriminator depth.

### 2.4. Performance of the Newly Developed Algorithm and Its Comparison with Previous Algorithms

**Figure 5:**
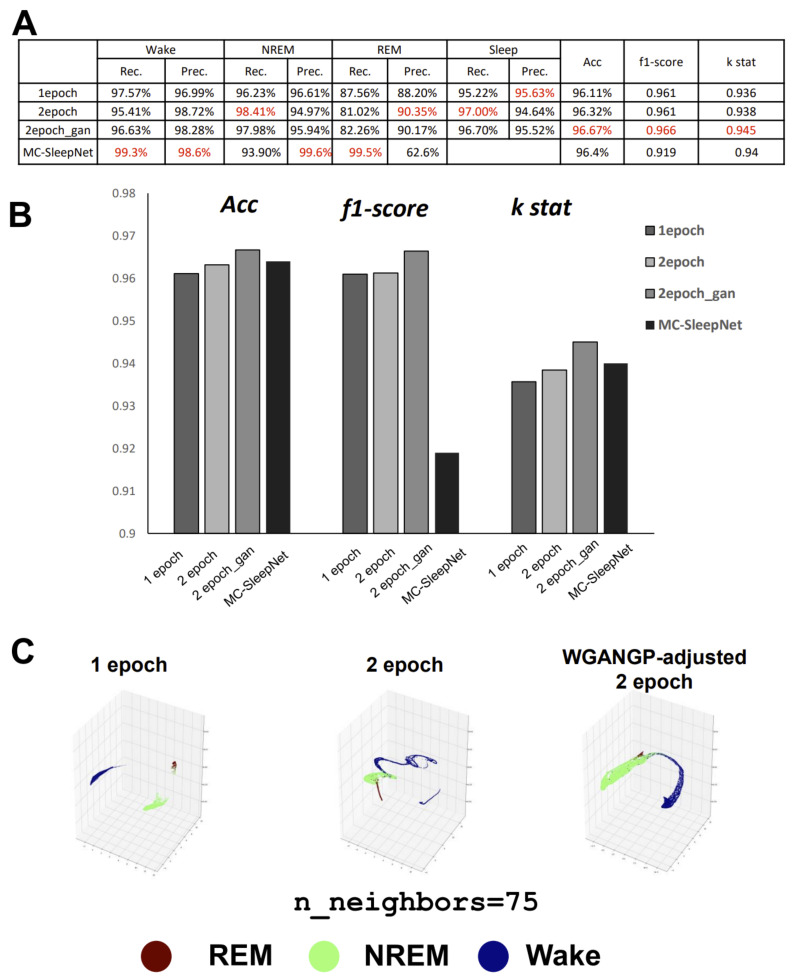
Performance of image-based sleep classification. (**A**) Scoring performance on Tsukuba-14 datasets compared with the original MC-SleepNet algorithm. Overall evaluation by three scales of accuracy, F1 score, and Cohen’s κ shows an improved performance with the additional one epoch and the assistance of the GAN-generated fake REM images. The scaled data of the MC-SleepNet are from the original work. The red font represents the highest performance in each column. Left side show the specific dataset we used for training (**B**) Comparison bar graph of three parameters between different algorithms. (**C**) Visualization of the dense layer of the model using the UMAP clustering algorithms. The different visible clustering separations display the scoring performance, particularly for the REM stage.

After debugging our small dataset, we evaluated the model’s fitting performance on another dataset, comparing it with current advanced models such as MC-SleepNet. We thus created images using Tsukuba-14 datasets. As we expected that redundant information would be beneficial to discriminate the data in sleep-stage transition, we created both one- and two-epoch datasets. This strategy is regarded as an extremely simplified version of LSTM, in which the “short memory” has only one previous set of epoch data. We also increased the REM data using the WGAN-GP. We examined three datasets, namely the one- and two-epoch datasets and the WGAN-GP-adjusted two-epoch dataset. Overall, our model performed almost as well, or even slightly better, in terms of accuracy and Cohen’s *κ* compared with MC-SleepNet (Figure 5A,B). The huge improvement in the F1 score is thought have benefited from the higher recall of REM. The WGAN-GP adjustment with fake REM images increased the overall accuracy. Even without this adjustment, the precision of REM on the two-epoch version maintained a high level, similar to that of MC-SleepNet on large-scale data. We believe this is because the spectral image features of REM are conducive to being identified.

To understand more intuitively how our classifier recognizes the interrelationships between the three stages within the model, we extracted the output information from both the first (128) and last (64) dense layers. The first dense layer is thought to collect all the feature information extracted from the CNN, while the last dense layer integrates all the information for the final classification. As that information is high-dimensional, to clearly see the distribution of each stage within the classification processing, a dimensionality reduction is necessary. UMAP (uniform manifold approximation and projection) is an effective dimension reduction and clustering tool. We embedded the output information of these dense layers into three components and visualized them in 3D space. We changed the parameters of n_neighbors from 5 to 100 (Supplementary Figure S1). The results were significant. When n_neighbors were set at 75 (Figure 5C), on the first dense layer, the three datasets showed variant distributions for the network and behaved consistently with scoring performances such as precision or recall. For the one-epoch dataset, the wake and sleep stages were completely separate, but many cases of NREM near to REM were observed, which is why the precision of sleep on the one-epoch dataset was the highest (Figure 5C, left). On the two-epoch dataset, REM exhibited a stick-like clustering and was connected with NREM, which matched the reality, as all phase-transition points of the sleep-activity cycle could be presented. NREM is always ahead of REM in a time series, so REM is only connected to NREM, but not wake (Figure 5C, center). The most exciting aspect was the performance on the WGAN-GP-adjusted dataset. As the fake REM data balanced the entire dataset, even the NREM and wake stages were in remarkable balance with each other, whereas the REM closely resembled a small branch growing on NREM, which was consistent with reality. Wake also became closer to REM, which could be regarded as the mid-wake points during sleep that often occur after REM (Figure 5C, right).

To evaluate how our fake REM images compared with the actual data, we performed visualization using the above processing method. On the first and last middle dense layers, with n_neighbors of 75, we observed that the fake REM and REM were completely combined and widespread in the UMAP 3D space (Supplementary Figure S2A,B). This distribution indicated two things: one is that the neural network successfully classifies real and fake images into one category; the other is that our fake REM has relatively high diversity, which needs to be handled with care when using DCGAN.

### 2.5. Effects of Different Epoch Length

**Figure 6:**
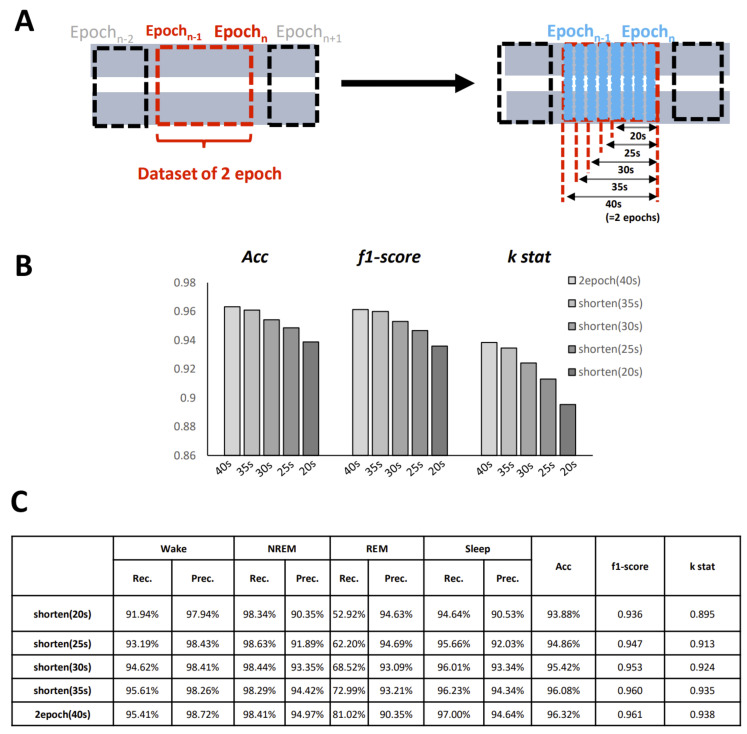
Effect of epoch length on the performance. (**A**) Schematic representation of the change in epoch length. (**B**) Comparison bar graph of three parameters between different epoch lengths. (**C**) Data table. Left side show the specific dataset with different length we used for training.

Another advantage of using images for judgment is that even images with missing information can also be recognized. To test the performance on non-standard datasets, we created images of different epoch lengths. As Figure 6A shows, we shortened the epoch length from 40 s (two epochs) to 20 s (one epoch) by 5 s, and then classified the images using our algorithm. The accuracy gradually decreased with the shortening of the epoch length, but even the shortest version (20 s) showed good performance (accuracy = 93.88%, *κ* *=* 0.8954) (Figure 6B,C). This indicated the robustness of our algorithm, and that it could be helpful in specific situations, such as with small-scale data breakage or short-term sampling instability. All of the shortened version results revealed a high grade of recall for the NREM and wake states, whereas the recall for REM showed an expected gradual decline with the shortening of length.

### 2.6. Performance on a Tiny Dataset

**Figure 7:**
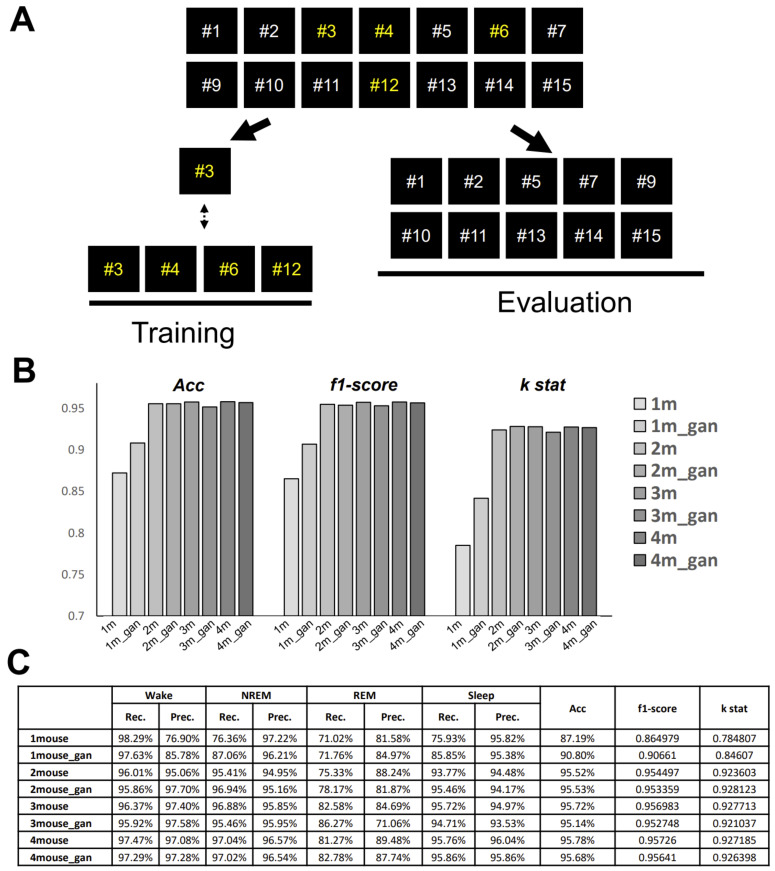
Performance on tiny datasets. (**A**) Schematic of the protocol to test the performance on tiny datasets. The 14-segment dataset was divided into two parts—segments 1 to 4 were used for training, and the remaining 10 segments were used as the evaluation dataset. Yellow font show the specific order number we used for training. (**B**) Comparison bar graph of three parameters under different conditions. (**C**) Data table. Left side show the specific dataset we used for training. ‘_gan’ represents the gan-created data has been added to the dataset for training.

Practically speaking, for most laboratory-level studies, performance on small-scale datasets is essential, as customizing the algorithm for different mouse strains to improve the accuracy of quantification is required. To investigate the performance on tiny datasets, we randomly selected single-mouse data and two-mice, three-mice, and four-mice data segments for use as the training dataset, and then applied the trained model to the other 10 mouse segments for use as the evaluation dataset. Although the results from using a single training segment dataset were not satisfactory, the performances of the two, three, and four training segment datasets were considered satisfactory, and the recall of REM also remained at an acceptable level (Figure 7). In addition, we expanded the REM data of the single-mouse dataset using WGAN-GP. This resulted in higher accuracy, but was still inferior to the two-mice datasets.

**Figure 8:**
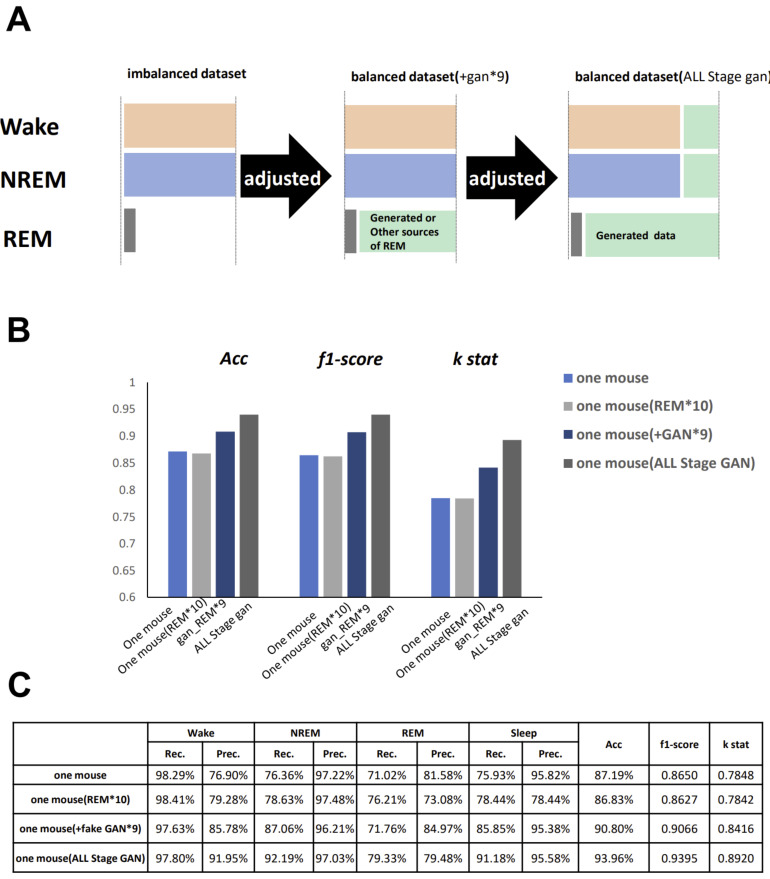
Expansion of all three-stage images by GAN. (**A**) Schematic representation of the protocol to create fake images for all three stages. (**B**) Comparison bar graph of three parameters between different conditions. (**C**) Data table. Left side show the specific dataset we used for training. ‘REM*10’ means that the data of REM is copied ten times than before. ‘gan_REM*9’ and ‘ALL_stage gan’ is described in A.

To improve the single-mouse data performance, we increased the number of images in all three stages by generating fake images of the wake, NREM, and REM stages (Figure 8A). On a tiny dataset of a single-mouse segment, the accuracy increased when we expanded all three-stage data with fake images (Figure 8B,C). Although the accuracy of 93.96% remained unsatisfactory, this value was still quite impressive for such a tiny dataset. In order to confirm if GAN is better than a simple increase in REM images, we tested another dataset with the simple addition of 10 times the number of REM images within a single mouse dataset, and the result was not improved at all.

### 2.7. Post-Prediction Filters for Further Improvement of Accuracy

In the final step, we found that when the accuracy was higher than 95%, certain points of error became conspicuous, and common errors could be corrected. For example, because the wake-stage EEG power spectrum was sometimes similar to the REM stage, some “REM stages” could be predicted within a continuous wake-stage period, particularly when the mouse was simply resting (i.e., not sleeping). To solve this problem, we designed a smoothing filter to remove atypical short REM periods from wake periods. The filters are also highly customizable if there is a necessity to adhere to special experimental requirements, and can be used together with other types of filters [15]. With these filters, the scoring performance was higher than previously (Supplementary Figure S3). It should be noted that this processing does not work on algorithms with low accuracy or on datasets with poor labeling quality, and can be counterproductive.

## 3. Discussion

GI-SleepNet, the novel image-based learning algorithm developed in this study, has several advantages over the conventional numerical data-based algorithm. First, the format of the data has excellent flexibility. In our case, one image has both raw EMG data and EEG frequency power spectrum data. EMG and EEG data differ between laboratories with different sampling rates and different data formats. Epoch lengths also differ among animal species. For human PSG, EOG should be included along with EMG and EEG. However, these differences do not matter once all the data are formatted as a single image. Thus, this method is readily applicable to any species and even outside of sleep research. Second, the image data that the machine learns has high interpretability. It is intuitively comprehensive, and each image contains sufficient visual information to classify it into three categories by researchers. Therefore, it is easy to create training datasets manually and to perform post-prediction analysis. Following automatic classification by machine, it is also easy to confirm the results and to find and resolve errors. Third, as image recognition is one of the most advanced fields in AI machine learning, it is easy to find sophisticated algorithms and to find recent progress. Therefore, we included the GAN method to adjust the REM data by producing fake REM images. Fourth, because the size of one set of data is limited to a 2D-image extent, and because the image-processing algorithms are optimized, our method requires relatively low computing power and a short processing time. Fifth, our method exhibits high accuracy with small datasets, making it useful in practice. It performed well on both our own dataset and those from different laboratories, even though these data were recorded using different types of acquisition equipment. This means that it can be easily reused on small datasets of specific strains or transgenic animals that may exhibit atypical EEG patterns. Thus, applying it to precious animal strains from which only a limited amount of data is available can also be advantageous to researchers.

**Figure 9:**
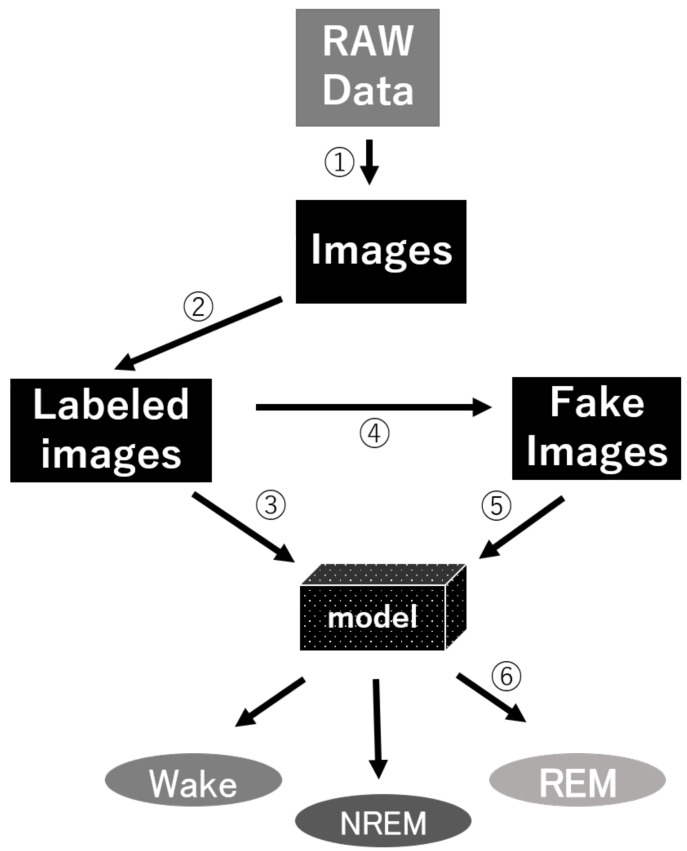
Our workflow diagram for GI-SleepNet: ① image production based on raw data, ② label the image data, ③ train the model, ④ generate fake data by GAN, ⑤ train the model with the generated images, and ⑥ make the prediction for new data.

We anticipate that researchers themselves will be able to customize our model. Researchers can use our workflow to create their own dataset and re-train a new model (Figure 9). To make our image-based scoring system easier to use, we developed a graphical user interface (GUI) for research purposes based on the Python binding GUI toolkit Tkinter (Supplementary Figure S4). Our GUI includes semi-automatic data-preprocessing, large-scale output of plotting images, neural network training, and prediction functions. We also released several trained h5 files for immediate use without a training dataset. The only action for individual researchers who wish to use our model is to create their own datasets and fit them to our shared network structure. Finally, the DCGAN-created images and forced filters based on our own dataset have also been packaged in the GUI. If this system can be used by a large number of researchers, we hope to collect considerably more data from different devices and further improve the noise resistance of the algorithm.

One important setting that should be considered is the length of the epoch. The selection of the epoch length is always difficult in sleep classification files. Conventionally, for humans, the epoch window is set to 30 s, and for mice, it is usually set to 20 s. However, there has been some commentary suggesting that a shorter epoch should be designed to improve the temporal resolution and thus evaluate time more efficiently. As our model is able to create an image for any length of epoch required, we call on others to create a shorter epoch dataset for further study.

Image-based scoring systems can be applied to identify not only sleep activity, but also other physiological and pathological events, such as epileptic seizures or preclinical Alzheimer’s disease symptoms. For example, one study was conducted that showed the differences in the prefrontal cortex EEG power spectrum during Y-maze tests between normal mice and Alzheimer’s disease models. A better understanding and design of experiments may be beneficial if creating image datasets of different performance groups in behavioral tests.

In this study, we only used two channels of biosignal from mice and obtained good results. We therefore expect that the program can be adapted to other systems. For example, small devices equipped with a single or a few channels of EEG are successfully used for many studies, including ours [16]. Those studies are good targets for our program, and can be used instead of laboratory PSG. It is expected that sleep studies can be facilitated at home and in broader settings [17]. As the GI-SleepNet is based on the relatively simple VGG-19-based algorithm, we believe that it is able to process the EEG/EMG more easily in order to measure sleep quality in the client terminal. The next step is to apply our model to human clinical PSG data. As the classification of human sleep is more complicated, we may increase the number of biosignals, such as EOG and heart rate, for further research [18].

Furthermore, in the future, particularly for specific experimental needs where more than three states need to be classified, our model is highly customizable. By modifying the units parameter of the output dense layer at the end of the model, it is possible to apply this model to other wider ranges of classification. In an ongoing study, we are applying this model to human clinical PSG datasets and classifying them into five states (NREM1, NREM2, NREM3, REM, and WAKE). The results so far are promising. Subsequently, for example, there is also the need for research to classify the middle transition state between two pure states. As the transition states are quite small in volume, we assume that the generation of fake data of certain transition states using our GAN model may promote performance if those transition states are recognized and labeled.

Our method does have some limitations. First, a priori knowledge is required to design images. Second, producing images is initially time consuming. In the case of our dataset, we used EMG and EEG signals of almost the same size. However, the ratio of sizes can be changed, which will affect accuracy. Nevertheless, to change the ratio, ensuring that all datasets contain all images for all epochs is required, a process that is time consuming and laborious. Despite these limitations, we believe that our novel algorithm will provide a versatile tool for future research in many fields. Third, a crucial point that needs to be resolved is the unquantified effect of EEG artifacts within the recording. In this study, we used a clear dataset with very few artifacts or noises (Tsukuba-14). Therefore, potential users should be aware of the fact that the performance against noise and artifacts needs to be experimented with in follow-up studies with other datasets containing artifacts. For the next step, we plan to artificially add random noise into the data to examine the artifact condition. As mentioned in the Materials and Methods section, some EEG artifacts featured within the recording could be absorbed by the model with the FFT as many artifacts occur during the wake stage and show an apparent low frequency. We expect that our model will be robust to those artifacts, but this should be examined in each dataset.

## 4. Materials and Methods

### 4.1. Training Hardware and Software

We used the following computer hardware and software environments:

#1: i7 9700K/GeForce RTX2070 super/64 GB, Anaconda Python 3.7, and TensorFlow 2.1.0;
#2: i7 9850HK/Quadro RTX3000/64 GB, Anaconda Python 3.7, and TensorFlow 2.1.0; and
#3: i9 10900KF/GeForce RTX3090/128 GB, Anaconda Python 3.8, and TensorFlow nightly version (tf-nightly gpu).

Computers #1 and #2 were mainly used for image dataset preparation and classification training. Computer #3 was used for image dataset preparation and GAN training to generate fake REM images. All PCs are Dell (Dell Technologies Japan Inc., Tokyo, Japan).

The classification training strategy involved using TensorBoard to monitor the metrics and to perform early stopping when necessary.

### 4.2. Sleep Data

Two separate mouse datasets were used in this study. We used our own datasets for preliminary algorithm development. EEG and EMG signals from 10- to 14-week old male C57/B6J mice (Clea Japan Inc., Tokyo, Japan) were recorded as described previously [19]. Briefly, the mice were anesthetized with isoflurane, and stainless-steel screws and wires were surgically implanted in the skull and the trapezius muscle, respectively. These served as electrodes and were connected to a microtip amplifier (Intan, RHD2216, 16-channel amplifier chip with bipolar inputs (Los Angeles, CA, USA), and an Open Ephys acquisition board for recording [20]. All data were recorded at least one week after the electrode implant surgery. The EEG and EMG signals from four mice were saved in a digital format file for further processing. To test the performance of our new algorithm, we used a small-scale dataset (Tsukuba-14 dataset) from a previous study [8]. The previous study also referred to recoding the EEG artifacts in the introduction of the dataset (not the Tsukuba-14 data). As we used the fast Fourier transform (FFT) power spectrum to replace the original raw waveform, similar to a special downsampling process, we predicted that the effect of the EEG artifacts would be partly reduced. However, it is important to note that the classification network and the GAN we used following that may have also absorbed some EEG artifact features within the recording.

The Tsukuba-14 dataset contained data segments from 14 mice, at 12 weeks old, with each segment containing four days of data (17,280 epochs of 20 s) for a single mouse.

### 4.3. Prediction and Calculations

The prediction model is presented in Figure 1 and Figure 2, and all the raw prediction results are shown in the Supplementary Table S1 (Microsoft Excel file). The values of the scoring valuation scale (accuracy, recall, F1-score, etc.) shown in the data table are the average values of the 14 (or the 10 for the tiny dataset valuation) individual mice. The customized calculation codes were performed based on the library Scikit-learn for Python. In general, a larger value on the scoring valuation scale means a better classification system performance.

## Data Availability

The datasets in this study are available from the corresponding author on request. The processing python code is provided from the following link: https://github.com/amakate/GaoTianXiang/blob/master/GiSleepNet_GUI_for_public_version1.ipynb (accessed on 10 October 2021).

## Supplementary Materials

The following are available online at https://www.mdpi.com/article/10.3390/clockssleep3040041/s1, Figure S1: Visualization of the dense layer of the model using the UMAP clustering algorithms: the distribution of all epoch data of the middle and last dense layers with various n_neighbor parameters set from 5 to 100, Figure S2: Visualization of the dense layer of the GAN model using the UMAP clustering algorithms. The distribution of all epoch data of the first middle dense layer (A) and the last middle dense layer (B) with n_neighbor parameters set at 75, Figure S3: Scoring performance with the forced correction filters: the filters can determine the epochs that we consider to be anomalies and fix those points. These exceptions include the REM epoch (for only 1–2 times) or the NREM epoch (for only 1–2 times) isolated over a long period of the wake stage. In these cases, they are corrected for the wake stage, Figure S4: The simply designed GUI is based on the standard Python interface Tkinter. It consists of three main functions: creating datasets based on customized requirements, training the labeled datasets, and predicting previous datasets. Currently, dat, edf, and csv data types can be processed. The DCGANs and forced automatic filter options are also open for users to create their own datasets for their experimental systems, Table S1: Confusion matrix of prediction results for all segment datasets.
