# Supplementary material for: GI-SleepNet: A Highly Versatile Image-Based Sleep Classification Using a Deep Learning Algorithm
Source: Clocks Sleep. 2021 Nov 1;3(4):581–97. doi: 10.3390/clockssleep3040041 (PMC8628800; doi:10.3390/clockssleep3040041)
Supplement: Supplementary file 1 [file clockssleep-03-00041-s001.zip › GI-SleepNet-MDPI-SupFigs-20211014.pdf]

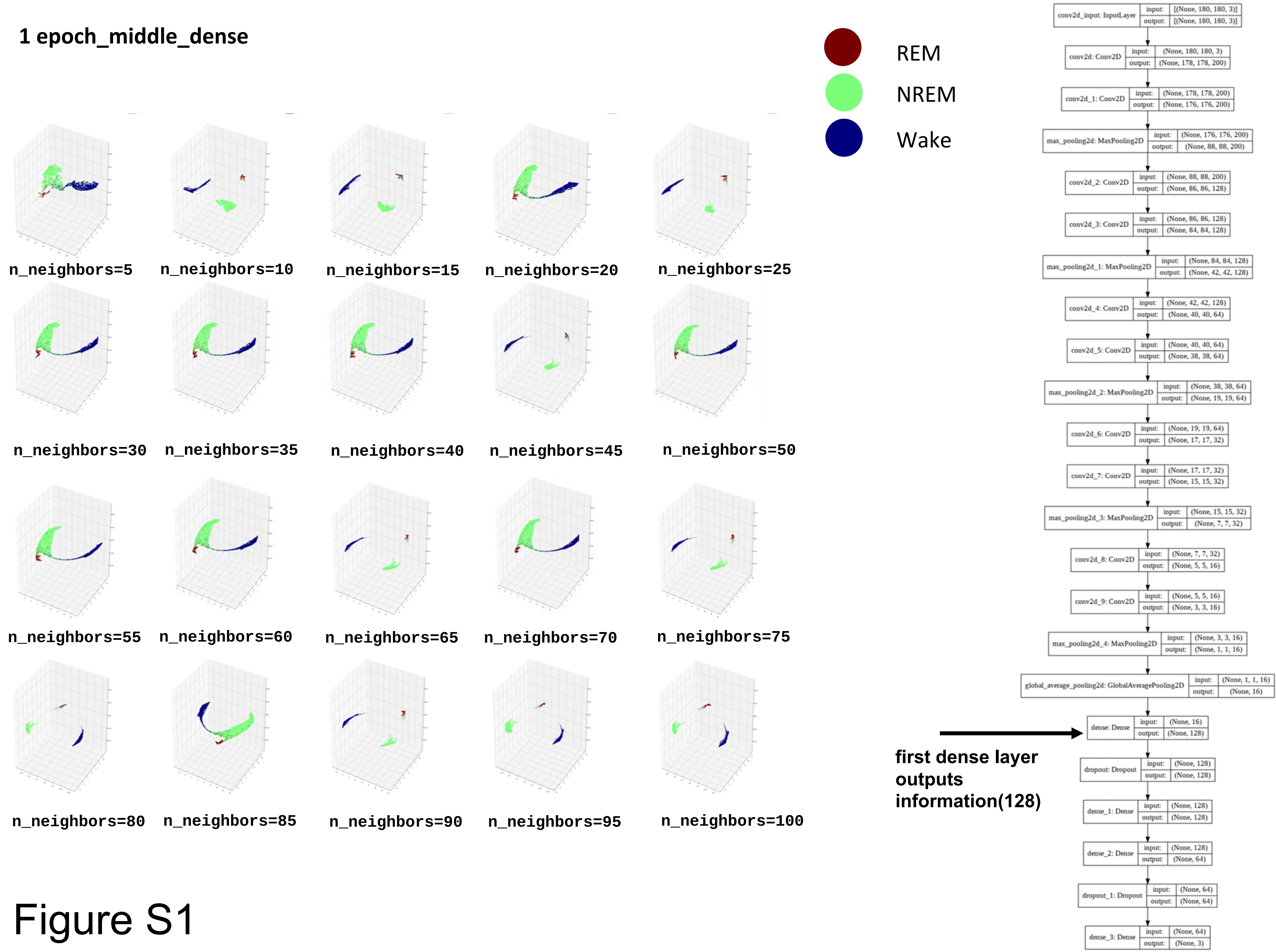
1 epoch_middle_dense
REM
NREM
Wake
n_neighbors=5
n_neighbors=10
n_neighbors=15
n_neighbors=20
n_neighbors=25
n_neighbors=30
n_neighbors=35
n_neighbors=40
n_neighbors=45
n_neighbors=50
n_neighbors=55
n_neighbors=60
n_neighbors=65
n_neighbors=70
n_neighbors=75
n_neighbors=80
n_neighbors=85
n_neighbors=90
n_neighbors=95
n_neighbors=100
first dense layer outputs information(128)
conv2d_input: InputLayer input: [(None, 180, 180, 3)] output: [(None, 180, 180, 3)]
conv2d: Conv2D input: (None, 180, 180, 3) output: (None, 178, 178, 200)
conv2d_1: Conv2D input: (None, 178, 178, 200) output: (None, 176, 176, 200)
max_pooling2d: MaxPooling2D input: (None, 176, 176, 200) output: (None, 88, 88, 200)
conv2d_2: Conv2D input: (None, 88, 88, 200) output: (None, 86, 86, 128)
conv2d_3: Conv2D input: (None, 86, 86, 128) output: (None, 84, 84, 128)
max_pooling2d_1: MaxPooling2D input: (None, 84, 84, 128) output: (None, 42, 42, 128)
conv2d_4: Conv2D input: (None, 42, 42, 128) output: (None, 40, 40, 64)
conv2d_5: Conv2D input: (None, 40, 40, 64) output: (None, 38, 38, 64)
max_pooling2d_2: MaxPooling2D input: (None, 38, 38, 64) output: (None, 19, 19, 64)
conv2d_6: Conv2D input: (None, 19, 19, 64) output: (None, 17, 17, 32)
conv2d_7: Conv2D input: (None, 17, 17, 32) output: (None, 15, 15, 32)
max_pooling2d_3: MaxPooling2D input: (None, 15, 15, 32) output: (None, 7, 7, 32)
conv2d_8: Conv2D input: (None, 7, 7, 32) output: (None, 5, 5, 16)
conv2d_9: Conv2D input: (None, 5, 5, 16) output: (None, 3, 3, 16)
max_pooling2d_4: MaxPooling2D input: (None, 3, 3, 16) output: (None, 1, 1, 16)
global_average_pooling2d: GlobalAveragePooling2D input: (None, 1, 1, 16) output: (None, 16)
dense: Dense input: (None, 16) output: (None, 128)
dropout: Dropout input: (None, 128) output: (None, 128)
dense_1: Dense input: (None, 128) output: (None, 128)
dense_2: Dense input: (None, 128) output: (None, 64)
dropout_1: Dropout input: (None, 64) output: (None, 64)
dense_3: Dense input: (None, 64) output: (None, 3)

Figure S1

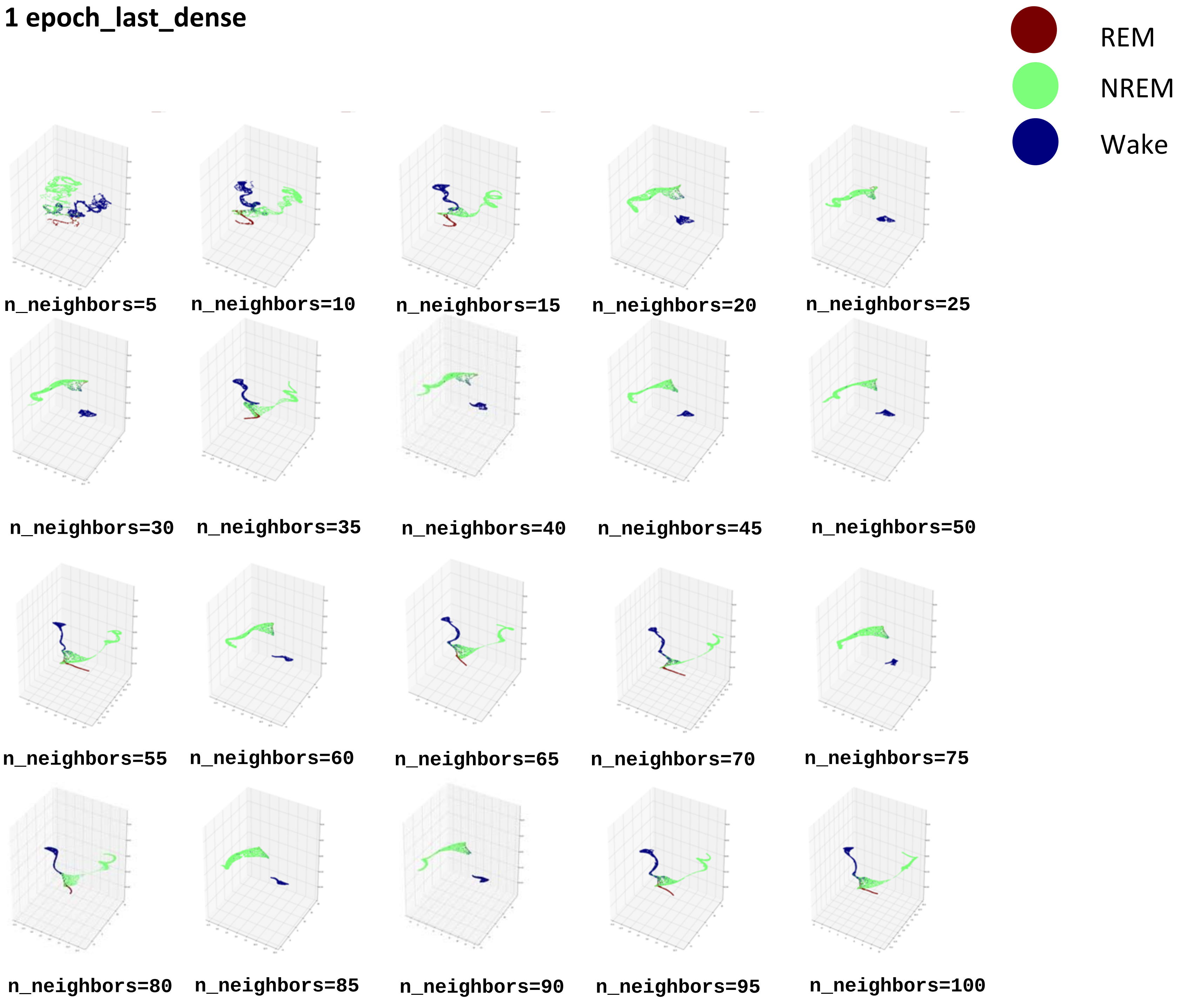

1 epoch_last_dense
REM
NREM
Wake
n_neighbors=5
n_neighbors=10
n_neighbors=15
n_neighbors=20
n_neighbors=25
n_neighbors=30
n_neighbors=35
n_neighbors=40
n_neighbors=45
n_neighbors=50
n_neighbors=55
n_neighbors=60
n_neighbors=65
n_neighbors=70
n_neighbors=75
n_neighbors=80
n_neighbors=85
n_neighbors=90
n_neighbors=95
n_neighbors=100

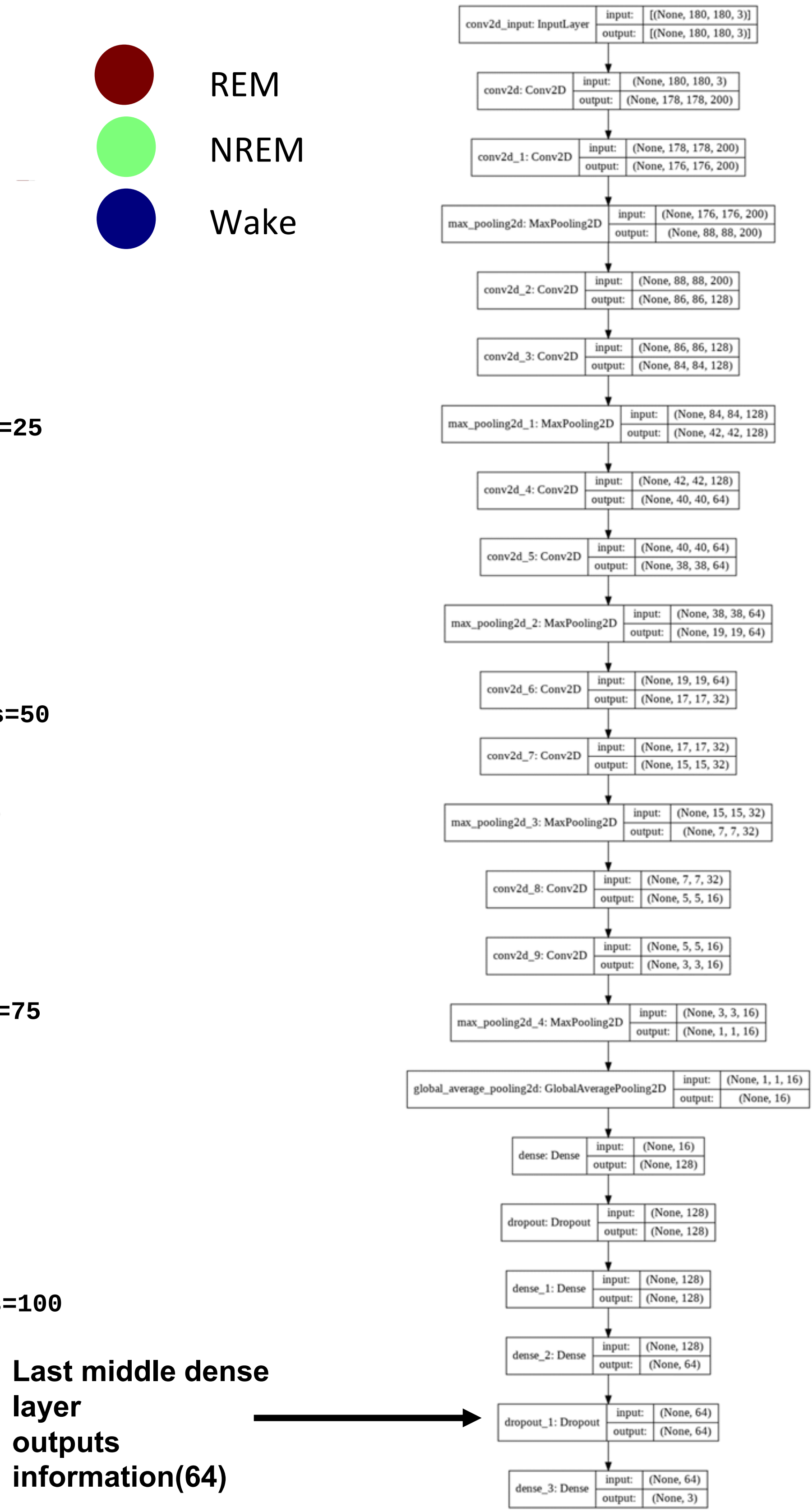

conv2d_input: InputLayer | input: [(None, 180, 180, 3)] | output: [(None, 180, 180, 3)]
conv2d: Conv2D | input: (None, 180, 180, 3) | output: (None, 178, 178, 200)
conv2d_1: Conv2D | input: (None, 178, 178, 200) | output: (None, 176, 176, 200)
max_pooling2d: MaxPooling2D | input: (None, 176, 176, 200) | output: (None, 88, 88, 200)
conv2d_2: Conv2D | input: (None, 88, 88, 200) | output: (None, 86, 86, 128)
conv2d_3: Conv2D | input: (None, 86, 86, 128) | output: (None, 84, 84, 128)
max_pooling2d_1: MaxPooling2D | input: (None, 84, 84, 128) | output: (None, 42, 42, 128)
conv2d_4: Conv2D | input: (None, 42, 42, 128) | output: (None, 40, 40, 64)
conv2d_5: Conv2D | input: (None, 40, 40, 64) | output: (None, 38, 38, 64)
max_pooling2d_2: MaxPooling2D | input: (None, 38, 38, 64) | output: (None, 19, 19, 64)
conv2d_6: Conv2D | input: (None, 19, 19, 64) | output: (None, 17, 17, 32)
conv2d_7: Conv2D | input: (None, 17, 17, 32) | output: (None, 15, 15, 32)
max_pooling2d_3: MaxPooling2D | input: (None, 15, 15, 32) | output: (None, 7, 7, 32)
conv2d_8: Conv2D | input: (None, 7, 7, 32) | output: (None, 5, 5, 16)
conv2d_9: Conv2D | input: (None, 5, 5, 16) | output: (None, 3, 3, 16)
max_pooling2d_4: MaxPooling2D | input: (None, 3, 3, 16) | output: (None, 1, 1, 16)
global_average_pooling2d: GlobalAveragePooling2D | input: (None, 1, 1, 16) | output: (None, 16)
dense: Dense | input: (None, 16) | output: (None, 128)
dropout: Dropout | input: (None, 128) | output: (None, 128)
dense_1: Dense | input: (None, 128) | output: (None, 128)
dense_2: Dense | input: (None, 128) | output: (None, 64)
dropout_1: Dropout | input: (None, 64) | output: (None, 64)
dense_3: Dense | input: (None, 64) | output: (None, 3)
Last middle dense layer outputs information(64)

Figure S1

2 epoch_middle_dense

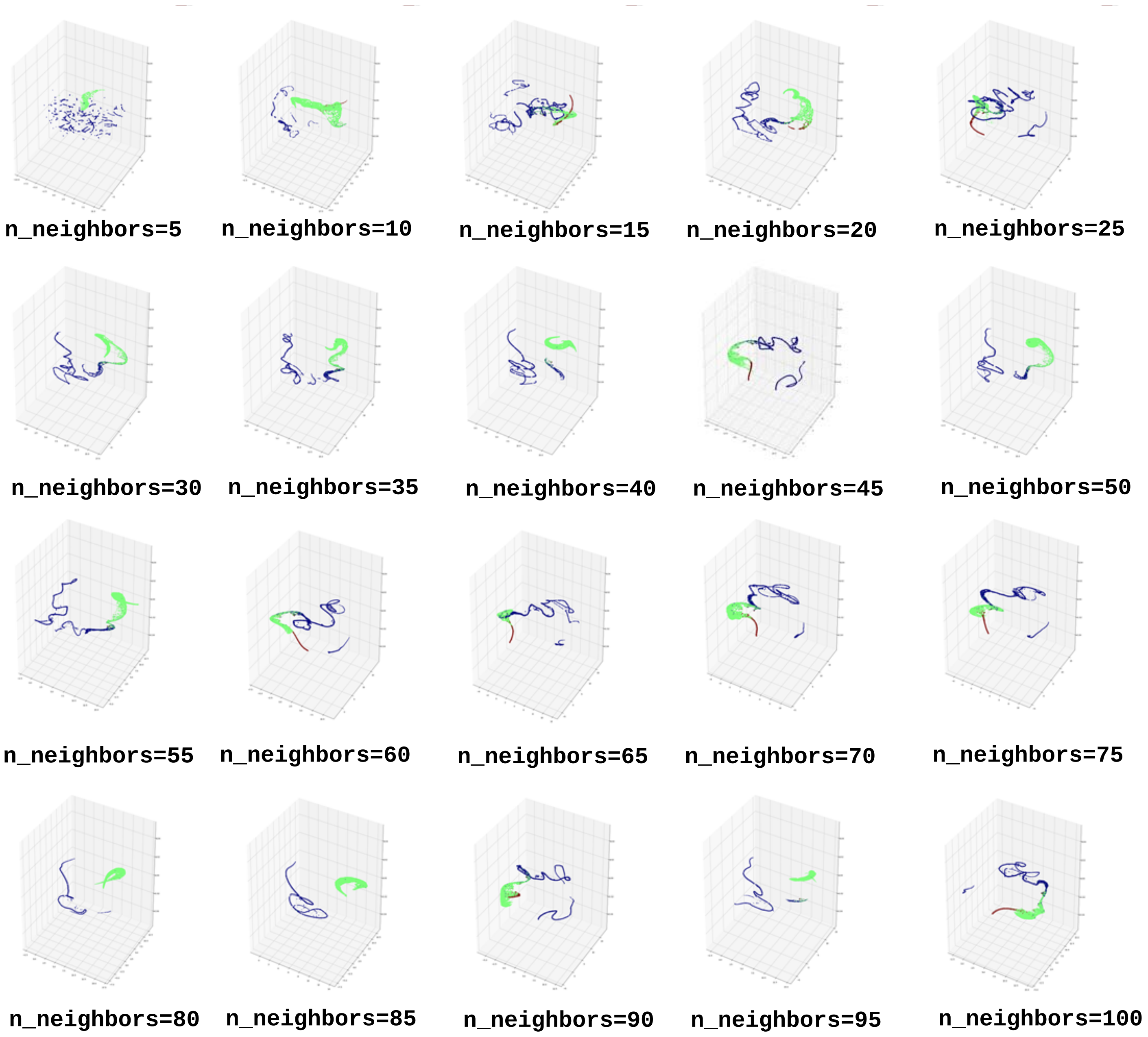

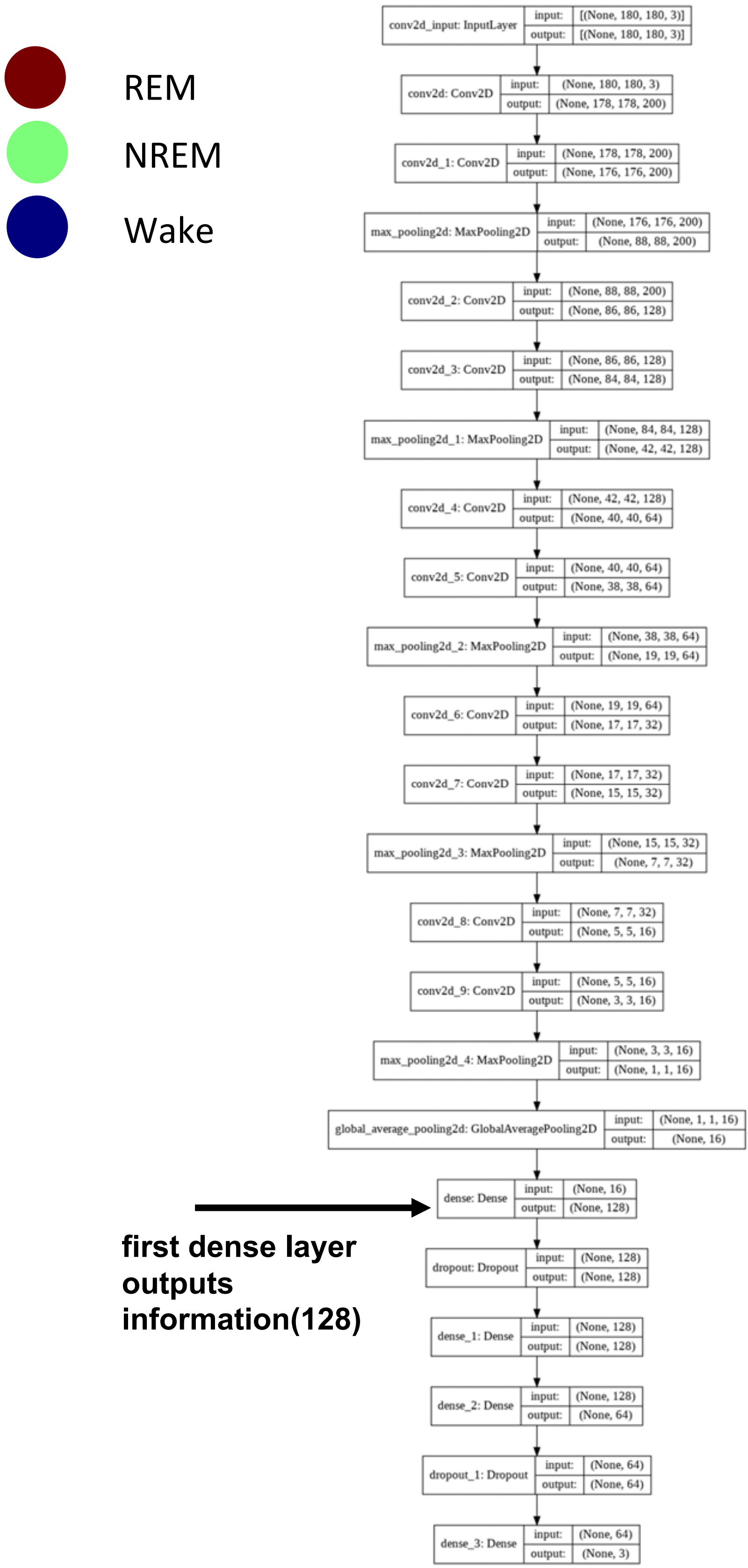

Figure S1

2 epoch_last_dense

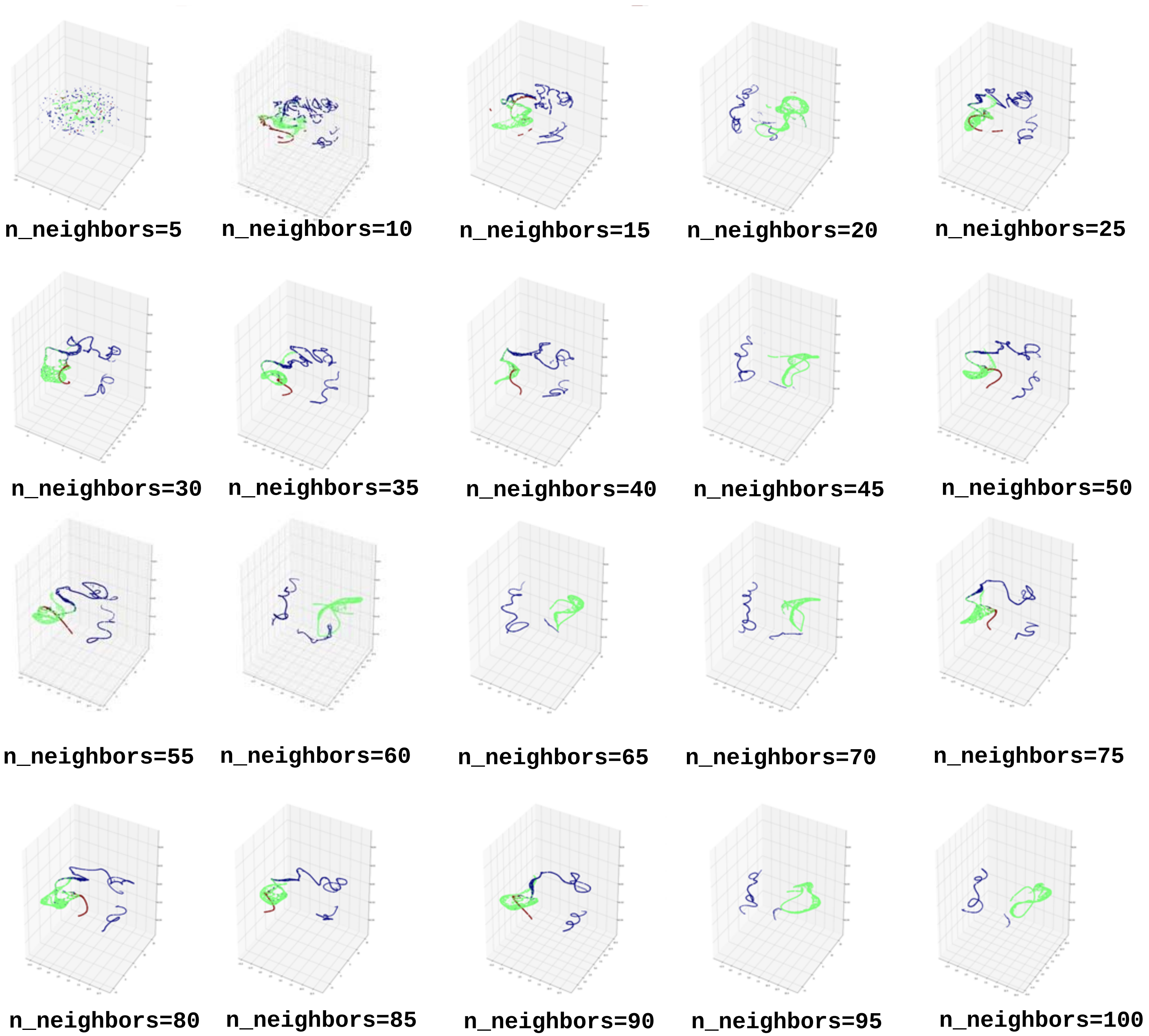

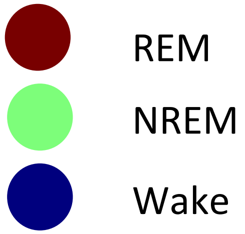

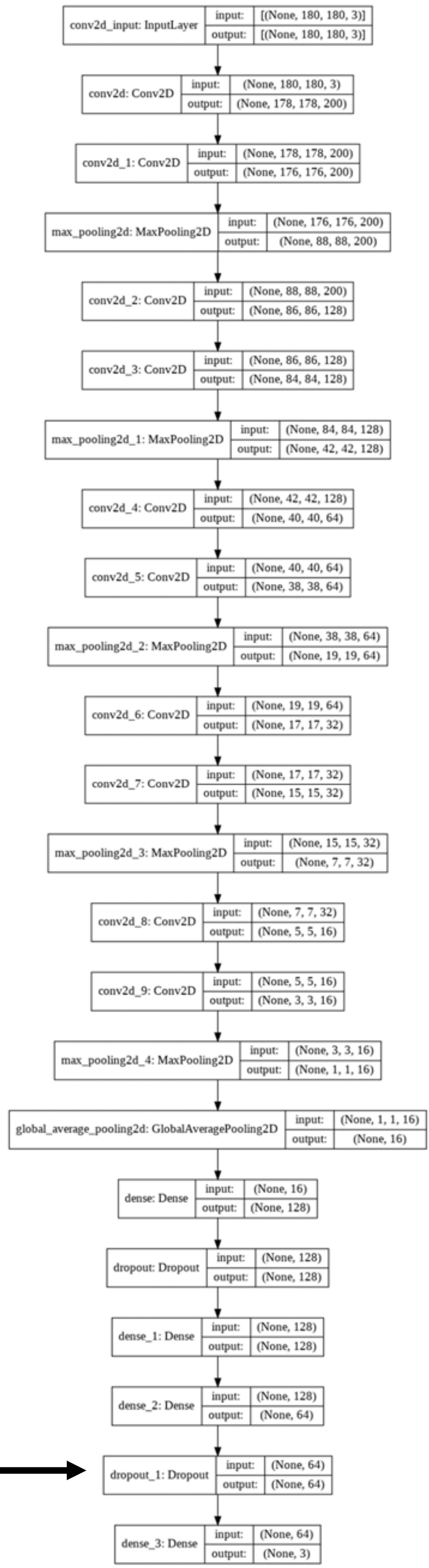

Figure S1

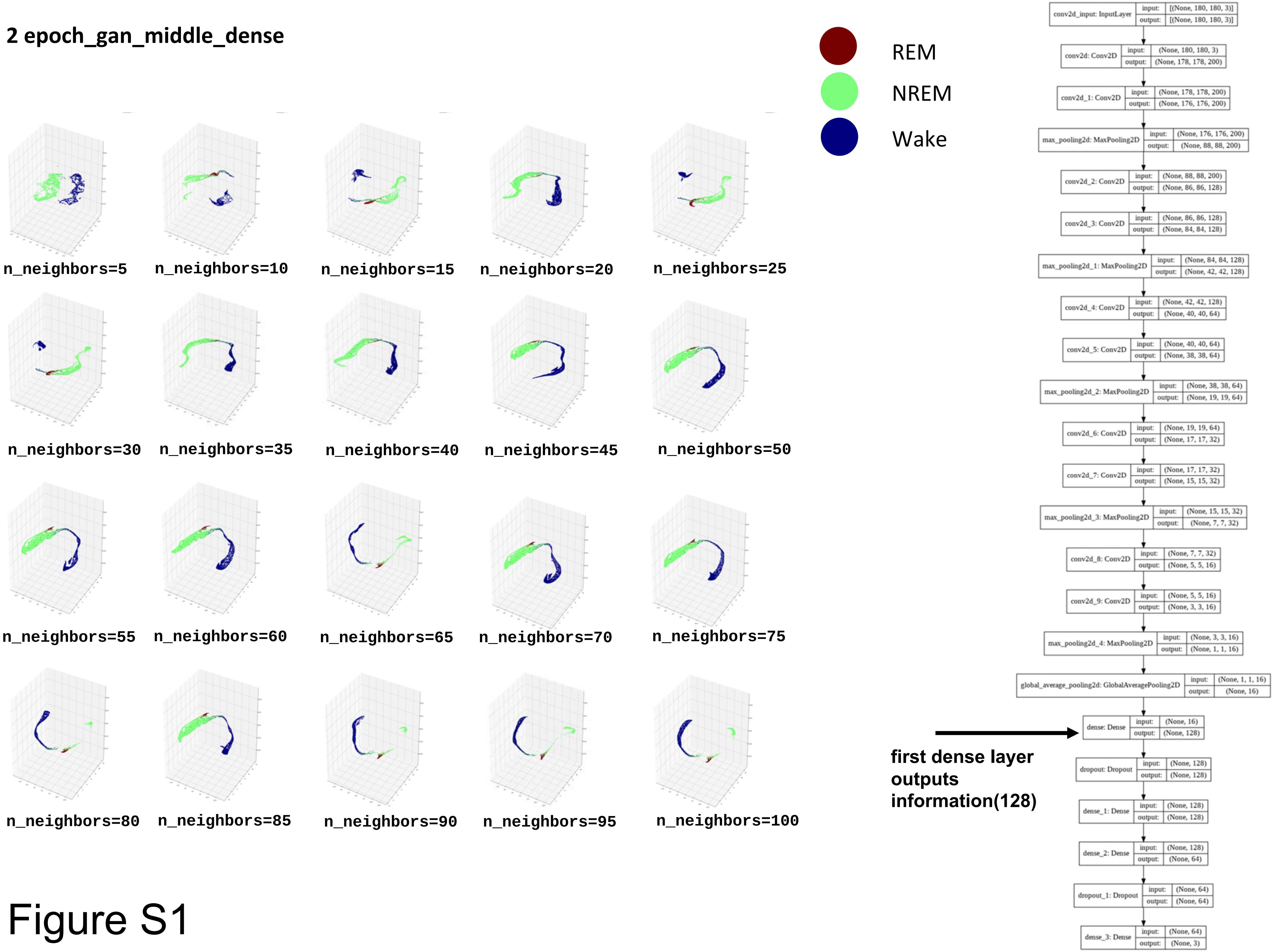
2 epoch_gan_middle_dense
REM
NREM
Wake
n_neighbors=5
n_neighbors=10
n_neighbors=15
n_neighbors=20
n_neighbors=25
n_neighbors=30
n_neighbors=35
n_neighbors=40
n_neighbors=45
n_neighbors=50
n_neighbors=55
n_neighbors=60
n_neighbors=65
n_neighbors=70
n_neighbors=75
n_neighbors=80
n_neighbors=85
n_neighbors=90
n_neighbors=95
n_neighbors=100
conv2d_input: InputLayer input: [(None, 180, 180, 3)] output: [(None, 180, 180, 3)]
conv2d: Conv2D input: (None, 180, 180, 3) output: (None, 178, 178, 200)
conv2d_1: Conv2D input: (None, 178, 178, 200) output: (None, 176, 176, 200)
max_pooling2d: MaxPooling2D input: (None, 176, 176, 200) output: (None, 88, 88, 200)
conv2d_2: Conv2D input: (None, 88, 88, 200) output: (None, 86, 86, 128)
conv2d_3: Conv2D input: (None, 86, 86, 128) output: (None, 84, 84, 128)
max_pooling2d_1: MaxPooling2D input: (None, 84, 84, 128) output: (None, 42, 42, 128)
conv2d_4: Conv2D input: (None, 42, 42, 128) output: (None, 40, 40, 64)
conv2d_5: Conv2D input: (None, 40, 40, 64) output: (None, 38, 38, 64)
max_pooling2d_2: MaxPooling2D input: (None, 38, 38, 64) output: (None, 19, 19, 64)
conv2d_6: Conv2D input: (None, 19, 19, 64) output: (None, 17, 17, 32)
conv2d_7: Conv2D input: (None, 17, 17, 32) output: (None, 15, 15, 32)
max_pooling2d_3: MaxPooling2D input: (None, 15, 15, 32) output: (None, 7, 7, 32)
conv2d_8: Conv2D input: (None, 7, 7, 32) output: (None, 5, 5, 16)
conv2d_9: Conv2D input: (None, 5, 5, 16) output: (None, 3, 3, 16)
max_pooling2d_4: MaxPooling2D input: (None, 3, 3, 16) output: (None, 1, 1, 16)
global_average_pooling2d: GlobalAveragePooling2D input: (None, 1, 1, 16) output: (None, 16)
dense: Dense input: (None, 16) output: (None, 128)
first dense layer outputs information(128)
dropout: Dropout input: (None, 128) output: (None, 128)
dense_1: Dense input: (None, 128) output: (None, 128)
dense_2: Dense input: (None, 128) output: (None, 64)
dropout_1: Dropout input: (None, 64) output: (None, 64)
dense_3: Dense input: (None, 64) output: (None, 3)

Figure S1

2 epoch_gan_last_dense

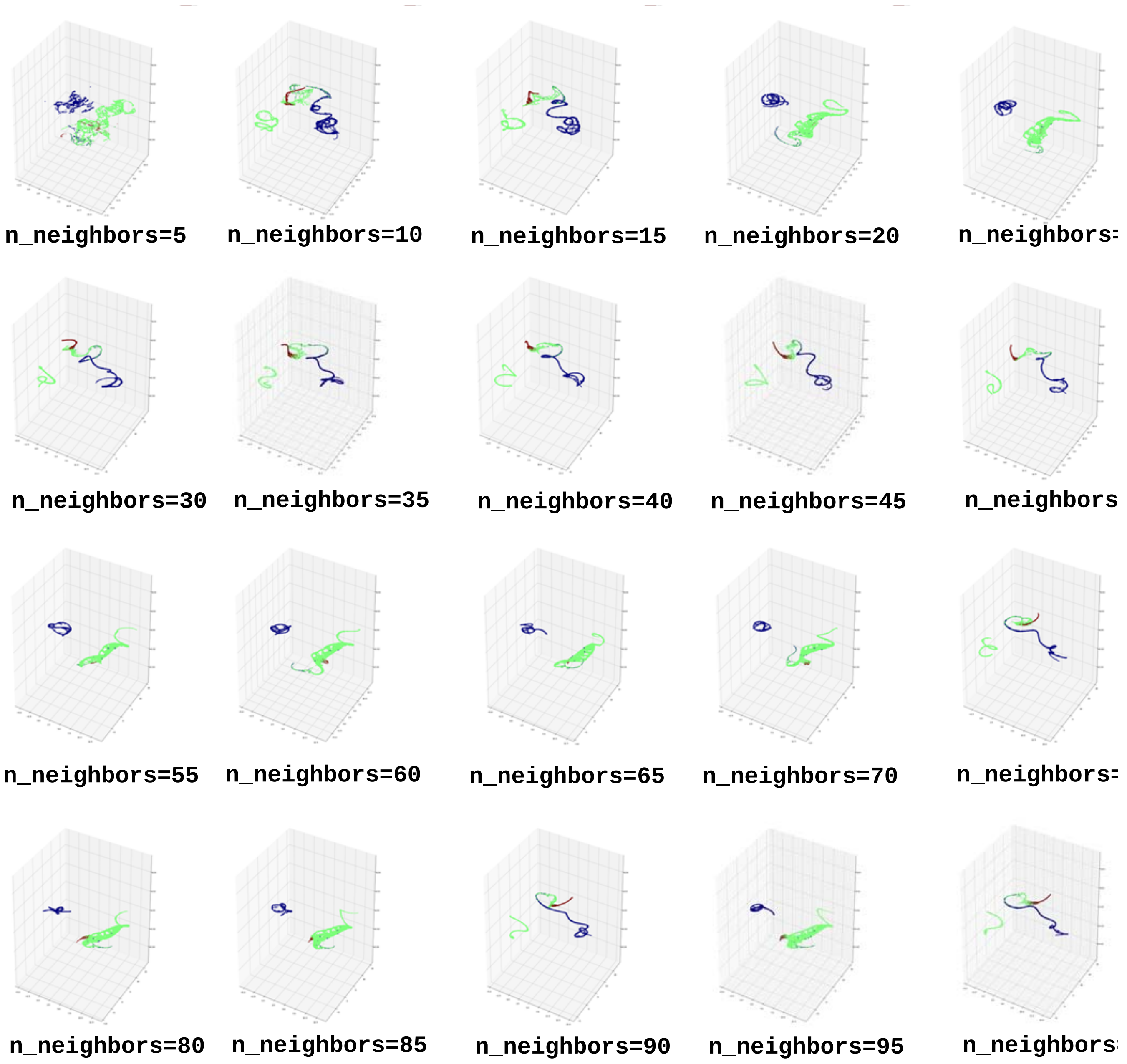

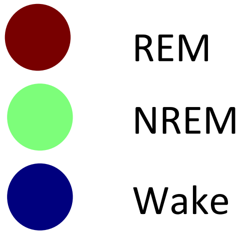

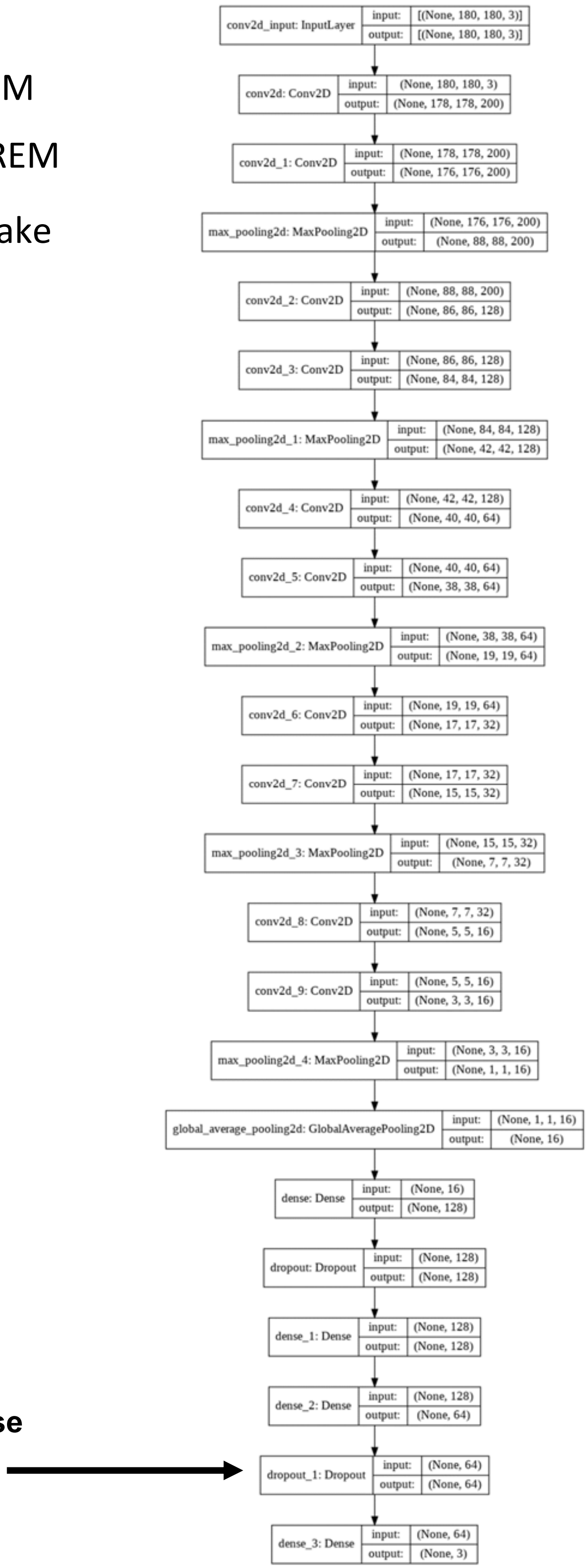

Last middle dense layer outputs information(64)

Figure S1

first middle dense layer outputs information(128)

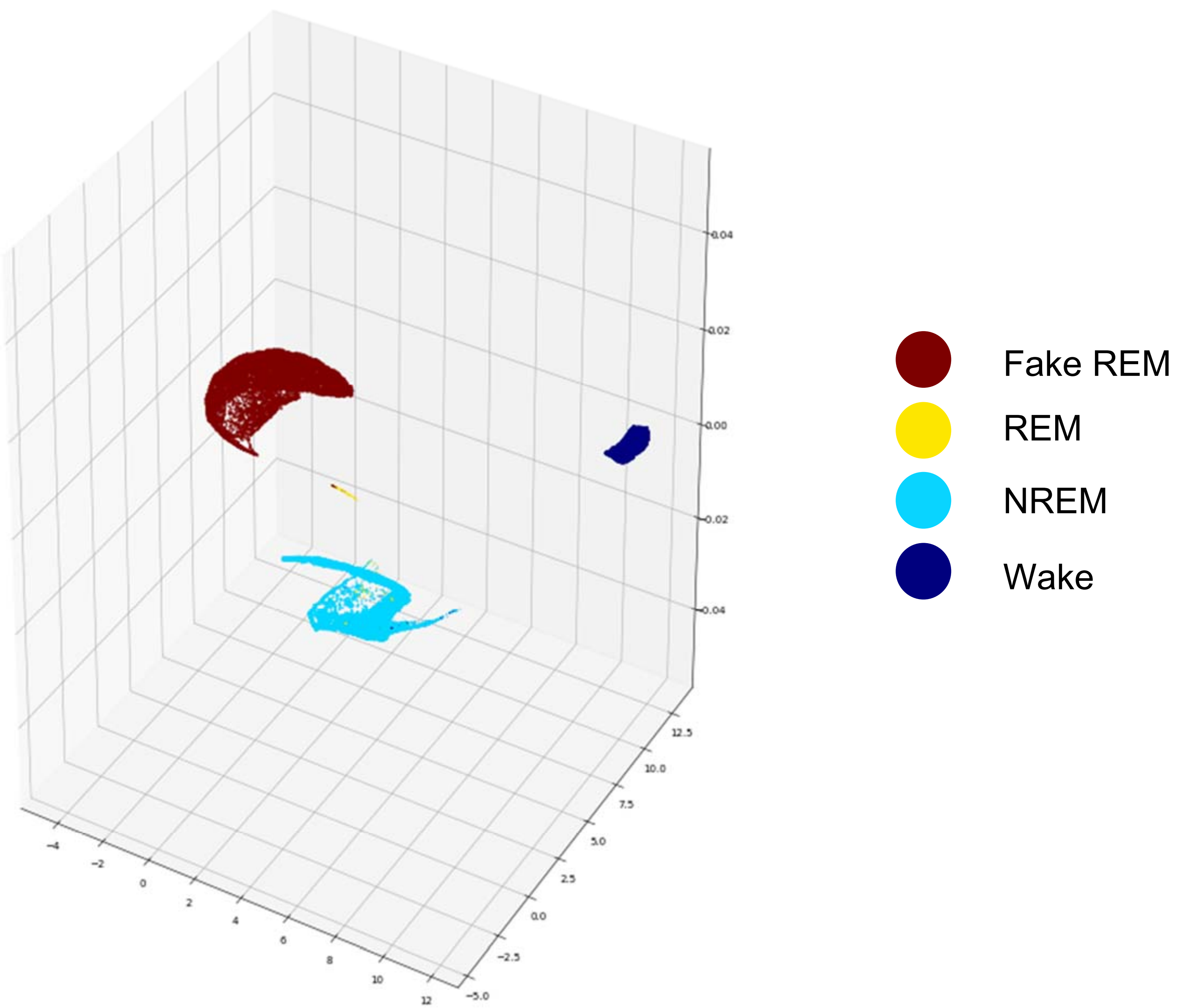

n_neighbors=75

Figure S2A

last middle dense layer outputs information(64)

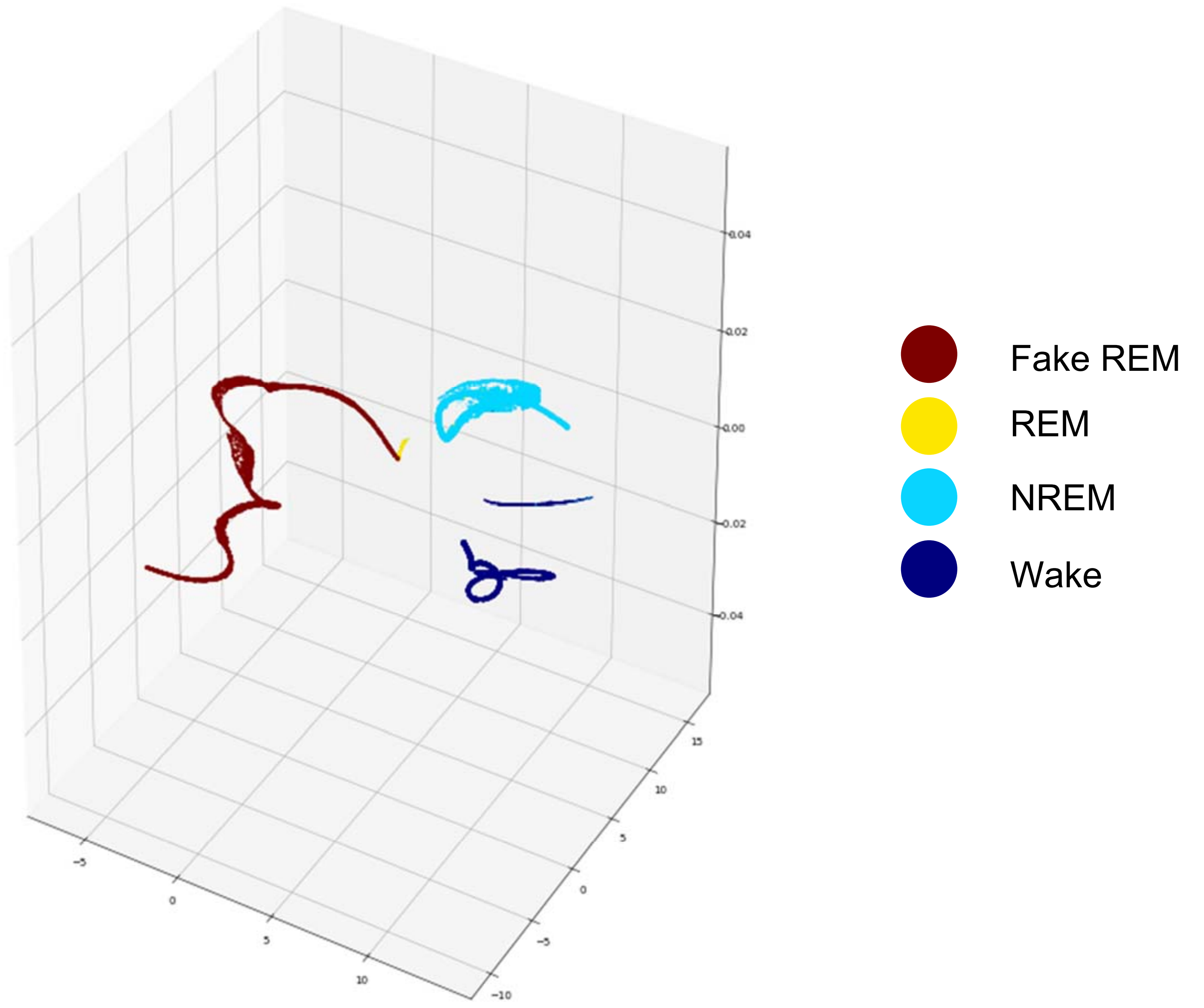

n_neighbors=75

Figure S2B

Scoring performance with the Forced correction filter

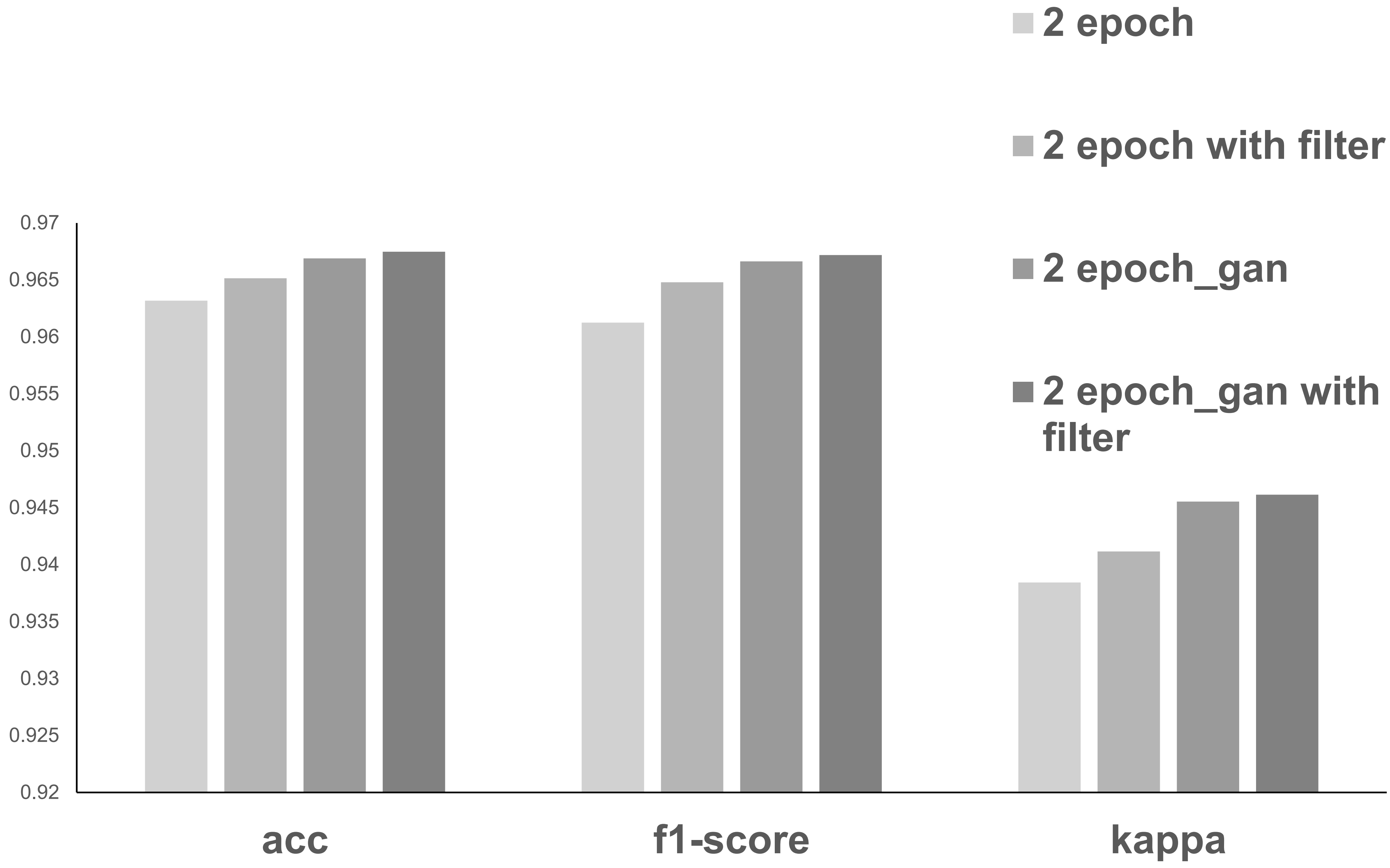

Figure S3

Simple GUI based on standard Python interface tkinter

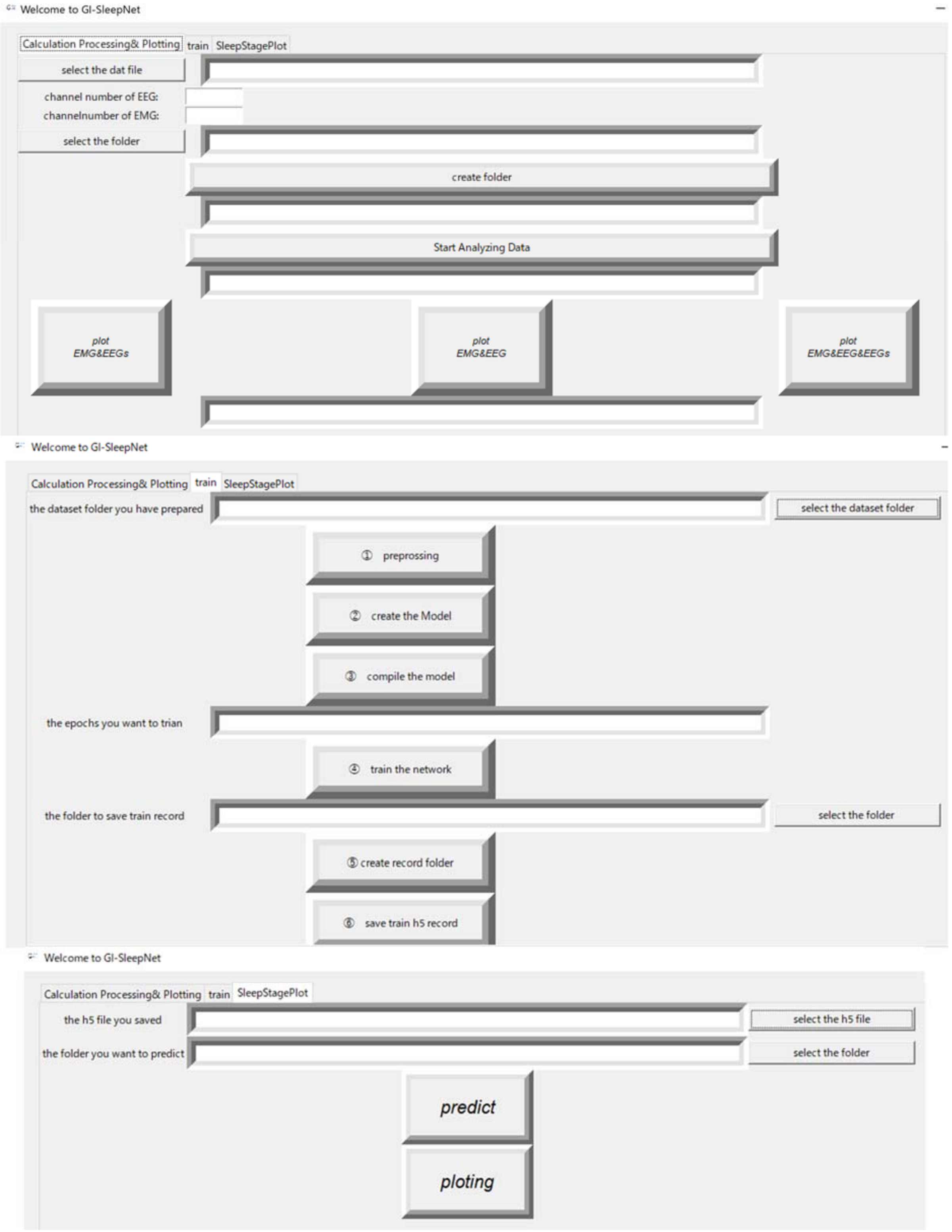

Figure S4
