# Supplementary material for: GI-SleepNet: A Highly Versatile Image-Based Sleep Classification Using a Deep Learning Algorithm
Source: Clocks Sleep. 2021 Nov 1;3(4):581–97. doi: 10.3390/clockssleep3040041 (PMC8628800; doi:10.3390/clockssleep3040041)
Supplement: Supplementary file 1 [file clockssleep-03-00041-s001.zip › GI-SleepNet-MDPI-SupTables-20210821.pdf]

one_epoch			2epoch			2epoch_filter			2epoch_gan			2epoch_gan_f		
#1			#1			#1			#1			#1		
7339	245	0	7159	426	0	7203	382	0	7230	354	1	7260	324	1
322	8457	69	87	8726	35	104	8709	35	141	8675	32	163	8653	32
27	204	6115	2	183	661	2	183	661	3	166	677	3	166	677
#2			#2			#2			#2			#2		
6380	208	1	6235	351	4	6285	301	4	6312	271	7	6345	238	7
405	9200	210	103	9616	96	120	9599	96	191	9523	101	217	9497	101
48	116	710	2	144	728	2	144	728	6	125	743	6	125	743
#3			#3			#3			#3			#3		
7544	220	13	7353	411	14	7410	354	14	7495	272	11	7534	233	11
252	8261	104	82	8492	43	89	8485	43	143	8433	41	149	8427	41
11	186	687	2	205	677	2	205	677	3	190	691	3	190	691
#4			#4			#4			#4			#4		
6555	255	8	6348	466	4	6437	377	4	6477	336	5	6533	280	5
333	8961	240	204	9241	90	231	9214	90	237	9220	78	252	9205	78
15	118	793	2	133	791	2	133	791	4	129	793	4	129	793
#5			#5			#5			#5			#5		
7366	288	5	7192	462	5	7295	359	5	7294	360	5	7362	292	5
269	8187	246	117	8471	114	131	8457	114	158	8418	126	177	8399	126
18	103	796	3	111	803	3	111	803	5	95	817	5	95	817
#6			#6			#6			#6			#6		
7321	168	4	7129	362	2	7295	359	5	7248	241	4	7290	200	3
102	8820	73	41	8914	40	131	8457	114	62	8902	31	83	8881	31
17	164	609	7	178	605	3	111	803	11	174	605	13	171	606
#7			#7			#7			#7			#7		
6663	108	7	6558	217	3	6593	182	3	6607	169	2	6633	143	2
171	9427	149	53	9636	59	59	9630	59	73	9612	63	82	9603	63
12	52	688	0	78	674	0	78	674	3	78	671	2	78	672
#9			#9			#9			#9			#9		
7504	180	3	7508	176	3	7536	149	2	7545	140	2	7560	125	2
58	8754	66	274	8587	18	302	8559	18	310	8546	23	337	8519	23
11	211	490	114	207	391	128	201	383	45	248	419	45	246	421
#10			#10			#10			#10			#10		
7774	231	1	7639	360	7	7713	286	7	7725	276	5	7778	223	5
140	8131	158	37	8305	88	42	8300	88	72	8265	93	82	8255	93
6	112	724	1	116	725	1	116	725	1	107	734	1	107	734
#11			#11			#11			#11			#11		
6991	111	13	6862	242	11	6903	201	11	6951	150	14	6977	124	14
96	9008	81	35	9141	10	67	9109	10	55	9113	18	93	9075	18
31	168	778	14	215	748	17	211	749	17	196	764	23	191	763
#12			#12			#12			#12			#12		
8295	66	4	8083	270	12	8135	218	12	8149	202	14	8188	163	14
459	7522	150	28	8006	98	33	8001	98	78	7948	106	92	7934	106
6	28	747	0	55	726	0	55	726	0	45	736	0	45	736
#13			#13			#13			#13			#13		
6519	163	2	6283	400	2	6344	339	2	6421	261	3	6455	227	3
107	9912	99	17	10013	88	58	9972	88	40	9971	107	84	9927	107
1	125	350	0	95	381	0	95	381	0	96	380	0	96	380
#14			#14			#14			#14			#14		
6992	123	4	6802	315	3	6842	275	3	6895	223	2	6918	200	2
86	9004	146	25	9186	25	76	9135	25	50	9147	39	104	9093	39
5	114	804	0	134	789	0	134	789	2	103	818	2	103	818
#15			#15			#15			#15			#15		
7267	74	2	7138	200	5	7170	168	5	7203	136	4	7225	114	4
107	9129	108	25	9221	98	50	9196	98	33	9235	76	57	9211	76
7	173	411	1	141	450	1	141	450	3	148	441	3	148	441

20s			25s			30s			35s		
#1			#1			#1			#1		
7007	578	0	7058	527	0	7123	462	0	7170	415	0
137	8687	24	127	8698	23	127	8686	35	148	8672	28
23	391	432	15	368	463	10	286	550	9	248	589
#2			#2			#2			#2		
6042	548	0	6110	480	0	6180	410	0	6228	361	1
208	9545	62	175	9585	55	174	9567	74	192	9546	77
18	322	534	14	276	584	12	234	628	9	204	661
#3			#3			#3			#3		
7165	608	5	7268	502	8	7357	413	8	7437	332	9
134	8465	18	113	8486	18	129	8455	33	145	8445	27
12	465	407	8	400	476	8	334	542	7	279	598
#4			#4			#4			#4		
6139	679	0	6189	629	0	6317	501	0	6412	406	0
236	9262	37	212	9288	35	234	9255	46	256	9227	52
12	388	526	8	327	591	8	271	647	7	227	692
#5			#5			#5			#5		
6550	1105	4	6727	929	3	6943	712	4	7087	570	2
153	8505	44	122	8530	50	129	8503	70	149	8485	68
15	307	595	11	247	659	6	211	700	5	181	731
#6			#6			#6			#6		
6487	1002	4	6741	748	4	6955	536	2	7078	414	1
36	8953	6	26	8958	11	25	8952	18	51	8927	17
3	500	287	2	395	393	5	327	458	10	263	517
#7			#7			#7			#7		
6468	310	0	6496	281	1	6539	239	0	6575	202	1
108	9625	15	77	9643	28	76	9633	39	83	9629	36
10	322	420	8	219	525	5	182	565	4	142	606
#9			#9			#9			#9		
7442	243	2	7445	240	2	7486	198	3	7513	172	2
176	8697	6	141	8731	7	202	8669	8	236	8633	10
27	522	163	28	428	256	38	370	304	42	331	339
#10			#10			#10			#10		
7401	603	2	7501	502	3	7596	406	4	7665	340	1
115	8287	28	104	8279	47	88	8293	49	75	8299	56
12	316	514	9	219	614	7	188	647	3	167	672
#11			#11			#11			#11		
6597	518	0	6700	411	4	6806	306	3	6890	219	6
45	9140	1	35	9149	2	31	9151	4	52	9129	5
22	456	499	25	352	600	18	309	650	21	283	673
#12			#12			#12			#12		
7808	557	0	7900	464	1	8013	349	3	8100	258	7
200	7923	9	98	8017	17	88	8012	32	94	7989	49
17	235	529	5	120	656	2	98	681	3	80	698
#13			#13			#13			#13		
6106	578	1	6170	514	1	6270	413	2	6329	355	1
128	9960	30	76	10011	31	63	9991	64	55	9997	66
1	227	248	0	194	282	0	155	321	0	131	345
#14			#14			#14			#14		
6644	477	2	6718	401	1	6816	303	1	6870	249	1
64	9159	13	45	9181	10	39	9176	21	40	9174	22
15	347	561	12	269	642	7	223	693	4	179	740
#15			#15			#15			#15		
6864	478	1	6979	362	2	7075	267	1	7142	200	1
63	9256	25	38	9276	30	38	9256	50	45	9245	54
5	326	261	2	307	283	2	239	351	4	207	381

1trainingdataset			2trainingdataset			3trainingdataset			4trainingdataset			3trainingdataset_gan			4trainingdataset_gan			2trainingdataset_gan			1mouse#3_gan			1m#3_plus#12			1m#3_plus#all			1m#3_plus#gan1665			1m#3_plus#gan3881			1m_#3_REM_repeat			1m_#3_REM_repeat			1m_#3_REM_repeat			1m#3_GAN_WNR		
#1			#1			#1			#1			#1			#1			#1			#1			#1			#1			#1			#1			#1			#1			#1			#1		
7513	70	2	7327	257	1	7306	277	2	7309	276	0	7296	287	2	7369	216	0	7291	292	2	7442	134	9	7270	198	117	7434	82	69	7394	182	9	7467	95	23	7477	98	10	7505	74	6	7503	71	11	7484	100	1
1445	7320	83	356	8459	33	302	8509	37	442	8371	35	310	8492	46	554	8244	50	267	8543	38	1107	7688	53	447	7649	752	1186	6472	1190	835	7910	103	1426	7302	120	1184	7513	151	1598	7167	83	1297	7406	145	1261	7493	94
93	160	593	19	281	546	17	235	594	48	227	571	21	215	610	49	221	576	14	213	619	43	243	560	8	84	754	21	70	755	16	155	675	62	134	650	42	102	702	113	149	584	32	117	697	130	128	588
#2			#2			#2			#2			#2			#2			#2			#2			#2			#2			#2			#2			#2			#2			#2			#2		
6374	212	4	6446	141	3	6397	186	7	6392	195	3	6418	164	8	6385	201	4	6404	185	1	6425	153	12	6434	144	12	6343	205	42	6409	177	4	6469	99	22	6481	96	13	6457	124	9	6499	82	9	6361	222	7
1109	8493	213	779	8861	175	613	9016	186	490	9194	131	558	9098	159	508	9160	147	474	9206	135	1053	8620	142	1107	8446	262	720	8779	316	992	8665	158	1647	7872	296	1396	8041	378	2218	7384	213	2489	7105	221	733	8891	191
55	131	688	57	104	713	44	83	747	42	132	700	54	96	724	35	114	725	21	117	736	36	126	712	36	99	739	24	95	755	40	107	727	52	45	777	43	35	796	65	75	734	70	85	719	37	103	734
#3			#3			#3			#3			#3			#3			#3			#3			#3			#3			#3			#3			#3			#3			#3			#3		
7569	197	12	7621	150	7	7616	152	10	7608	161	9	7576	190	12	7607	163	8	7561	203	14	7582	182	14	7600	168	10	7539	226	13	7584	184	10	7578	176	24	7589	174	15	7548	213	17	7602	165	11	7640	127	11
133	8428	56	154	8386	77	135	8402	80	135	8446	36	144	8402	71	83	8496	38	134	8406	77	164	8405	48	192	8359	66	126	8450	41	139	8409	69	221	8264	132	196	8295	126	194	8359	64	222	8341	54	197	8365	55
1	48	835	1	83	800	0	50	834	2	78	804	4	47	833	2	63	819	2	92	790	2	157	725	1	96	787	4	60	820	1	52	831	2	72	810	1	23	860	1	32	851	0	1	883	6	84	794
#4			#4			#4			#4			#4			#4			#4			#4			#4			#4			#4			#4			#4			#4			#4			#4		
6757	58	3	6643	171	4	6688	127	3	6673	140	5	6644	172	2	6671	147	0	6713	103	2	6782	36	0	6755	35	28	6567	14	237	6768	48	2	6763	28	27	6802	11	5	6771	41	6	6782	31	5	6754	60	4
3070	6286	179	2051	7333	151	282	9154	99	230	9233	72	247	9167	121	104	9358	73	1976	7432	127	4209	5248	78	4438	4888	209	876	1150	7509	5751	3693	91	4686	4676	173	5919	3381	235	3939	5446	150	4548	4797	190	5459	3973	103
154	55	717	131	88	707	5	48	873	7	54	865	0	28	898	4	52	870	149	95	682	304	69	553	201	46	679	2	1	923	369	48	509	273	29	624	355	9	562	153	62	711	213	60	653	311	55	560
#5			#5			#5			#5			#5			#5			#5			#5			#5			#5			#5			#5			#5			#5			#5			#5		
7642	15	2	7196	459	4	7232	418	9	7262	391	6	7267	336	56	7251	399	9	7176	434	49	7592	63	4	6292	191	1176	0	0	7659	7480	171	8	7590	29	40	7615	34	10	7644	12	3	7597	51	11	7500	151	8
6523	1971	208	222	8380	100	238	8075	389	527	7858	317	282	6908	1512	452	7856	394	223	7868	611	3666	4912	124	124	1887	6691	0	0	8702	1082	7532	88	3053	2181	3468	6307	2197	198	7093	1492	117	6085	2243	374	838	7608	256
574	11	332	11	222	684	17	62	838	40	48	829	13	11	893	27	43	847	8	50	859	359	71	487	3	14	900	0	0	917	68	226	623	215	68	634	570	12	335	619	12	286	552	11	354	72	87	758
#6			#6			#6			#6			#6			#6			#6			#6			#6			#6			#6			#6			#6			#6			#6			#6		
7136	350	7	6275	1199	19	6828	587	78	7401	91	1	6667	553	273	7404	85	4	6421	828	244	6916	423	154	4738	719	2036	0	0	7493	6521	968	4	7009	273	211	7124	352	17	7255	228	10	6971	491	31	7311	177	5
369	8507	119	89	8888	18	129	8715	151	65	8887	43	136	8018	841	40	8919	36	111	8202	682	308	8664	23	73	8525	397	0	0	8995	260	8720	15	348	8524	123	317	8576	102	405	8534	56	292	8373	330	426	8467	102
42	120	628	15	369	406	22	147	621	12	37	741	17	29	744	6	30	754	12	65	713	34	358	398	3	199	588	0	0	790	27	552	211	34	407	349	35	151	604	50	181	559	31	101	658	50	216	524
#7			#7			#7			#7			#7			#7			#7			#7			#7			#7			#7			#7			#7			#7			#7			#7		
6709	60	9	6623	151	4	6583	187	8	6634	142	2	6585	182	11	6638	137	3	6590	185	3	6578	177	23	6443	230	105	6593	67	118	6550	213	15	6611	112	55	6638	117	23	6714	54	10	6657	107	14	6668	103	7
1492	8105	151	174	9500	74	105	9574	69	157	9532	59	104	9527	117	179	9480	89	106	9542	100	949	8705	94	249	8419	1080	520	6115	3113	746	8837	165	1420	7852	476	1142	8333	273	2976	6639	133	1205	8354	189	684	8928	136
24	53	675	5	114	633	5	92	655	21	94	637	3	59	690	17	70	665	8	96	648	7	102	643	1	23	728	0	17	735	5	70	677	9	28	715	15	30	707	87	49	616	13	57	682	69	79	604
#9			#9			#9			#9			#9			#9			#9			#9			#9			#9			#9			#9			#9			#9			#9			#9		
7490	186	11	7427	251	9	7426	257	4	7414	265	8	7377	291	19	7416	260	11	7389	292	6	7419	258	10	7451	228	8	7411	268	8	7375	299	13	7454	208	25	7568	108	11	7503	173	11	7567	111	9	7439	231	17
271	8532	76	195	8632	52	118	8712	49	92	8748	39	92	8732	55	94	8744	41	91	8738	50	275	8572	32	244	8538	8	129	8660	90	165	8596	118	363	8416	100	890	7866	123	503	8306	70	858	7896	125	215	8569	95
30	186	496	44	231	437	26	212	474	26	241	445	12	192	508	18	240	454	12	258	442	16	283	413	21	167	524	20	199	493	16	183	513	23	147	542	111	98	503	30	198	484	76	152	484	31	152	529
#10			#10			#10			#10			#10			#10			#10			#10			#10			#10			#10			#10			#10			#10			#10			#10		
7866	126	14	7693	304	9	7647	349	10	7743	262	1	7613	376	17	7738	259	9	7628	371	7	7801	176	29	7577	324	105	7702	198	106	7726	260	20	7840	131	35	7810	172	24	7904	82	20	7831	148	27	7842	148	16
606	7628	196	154	8157	119	119	8206	105	154	8209	67	86	8220	124	153	8173	104	111	8210	109	464	7835	131	178	7704	548	351	6652	1427	358	7857	215	760	7395	275	469	7611	350	1033	7201	196	497	7637	296	408	7822	200
12	83	747	5	132	705	3	115	724	14	152	676	4	127	711	11	120	711	2	132	708	2	115	725	1	65	776	3	49	790	0	81	761	9	49	784	3	41	798	14	86	742	2	79	761	18	86	738
#11			#11			#11			#11			#11			#11			#11			#11			#11			#11			#11			#11			#11			#11			#11			#11		
7078	29	8	6948	161	6	6897	209	9	6931	18	4	6907	189	19	6919	185	11	6873	233	9	7005	92	18	6865	126	124	6856	28	231	6994	103	18	7058	29	28	7040	46	29	7086	18	11	7026	63	26	7010	86	19
3897	5213	76	141	9026	19	84	9084	18	75	9106	5	67	9078	41	64	9112	10	67	9105	14	2133	7018	35	273	6591	2322	496	1050	7640	2017	7109	60	3832	5256	98	2759	6201	226	5481	3651	54	3185	5881	120	597	8505	84
253	69	655	32	241	704	30	256	691	35	289	653	15	193	769	29	274	674	14	269	694	142	149	686	20	26	931	21	10	946	119	130	728	245	44	688	212	38	727	343	65	569	195	92	690	93	139	745
#12			#12			#12			#12			#12			#12			#12			#12			#12			#12			#12			#12			#12			#12			#12			#12		
8363	2	0	8268	94	3	8311	52	2	8286	78	1	8288	76	1	8303	58	4	8224	136	5	8358	4	3	8051	105	209	8101	16	248	8359	3	3	8360	1	4	8361	1	3	8365	0	0	8359	1	5	8344	11	10
7366	737	29	72	8018	42	55	8024	53	40	8053	39	90	7963	79	22	8086	24	36	8039	57	6966	1129	37	414	2648	5070	776	990	6366	6793	1291	48	7335	763	34	7057	989	86	7686	425	21	7100	973	59	5697	2362	73
688	1	92	0	20	761	1	31	749	0	29	752	2	12	767	0	32	749	0	43	738	645	5	131	1	0	780	0	0	781	541	10	230	690	1	90	674	2	105	699	3	79	654	5	122	459	18	304
#13			#13			#13			#13			#13			#13			#13			#13			#13			#13			#13			#13			#13			#13			#13			#13		
6621	59	5	6470	215	0	6409	274	2	6412	273	0	6350	325	10	6395	285	5	6332	348	5	6440	141	4	6555	112	18	6529	68	88	6493	189	3	6649	33	3	6587	83	15	6677	8	0	6580	97	8	6469	202	14
1503	8501	105	175	9897	46	108	9938	72	79	9978	61	72	9939	107	95	9971	52	75	9978	65	879	9188	51	568	8764	786	830	2717	6571	768	9226	124	2206	7800	112	928	8999	191	4286	5788	44	1186	8767	165	372	9609	137
82	105	289	5	193	278	4	157	315	0	142	334	1	131	344	2	173	301	0	177	299	30	182	264	6	26	444	3	0	473	39	114	323	121	53	302	21	68	387	193	67	216	25	92	359	11	114	351
#14			#14			#14			#14			#14			#14			#14			#14			#14			#14			#14			#14			#14			#14			#14			#14		
700	103	17	6933	182	5	6888	221	11	6939	173	8	6809	292	19	6891	216	13	6842	272	6	6920	185	15	6972	131	17	6937	145	38	6866	232	22	7003	89	28	6999	91	30	7077	27	16	6948	149	23	6963	137	20
430	8602	204	174	8993	69	129	8991	116	128	9062	46	72	9076	88	96	9069	71	96	9085	55	415	8699	122	405	8396	435	290	8264	682	227	8854	155	688	8227	321	748	7943	545	1267	7774	195	632	8392	212	359	8700	177
17	61	845	8	138	777	17	76	830	12	133	778	5	93	825	6	120	797	5	130	788	11	87	825	7	36	880	8	40	875	6	69	848	23	28	872	36	21	866	32	55	836	34	61	828	27	55	841
#15			#15			#15			#15			#15			#15			#15			#15			#15			#15			#15			#15			#15			#15			#15			#15		
7252	80	11	7046	291	6	7050	280	13	7252	88	3	7023	303	17	7244	96	3	7037	298	8	7129	200	14	6439	505	399	7039	130	174	7040	289	14	7162	154	27	7162	161	20	7269	64	10	7208	121	14	7236	99	8
199	8991	11	70	9195	79	43	9207	94	132	9134	78	37	9167	140	141	9110	93	42	9206	96	136	9126	82	28	8999	317	128	8577	639	71	9107	166	229	8841	274	149	8970	225	461	8737	146	225	8897	222	238	8973	133
10	154	428	2	235	355	1	211	380	9	191	392	1	165	426	7	180	405	0	201	391	5	225	362	0	128	464	0	79	513	3	150	439	4	116	472	6	104	482	27	148	417	5	132	455	23	181	388
